# The Efficacy of a Home Monitoring App for Children With Medical Complexity: Protocol for a Randomized Controlled Trial

**DOI:** 10.2196/108485

**Published:** 2026-10-22

**Authors:** Flory Nkoy, Bryan Stone, Carolyn Foster, Angela Zhu, Sara McCormick, Maya Wheeler, Peter Walsh, Connor Karwowski, Savannah East, Katherine Sward, Xiaoming Sheng, Carolyn Reynolds, Justin B Poll, Nancy Murphy

**Affiliations:** 1 Pediatrics, School of Medicine, University of Utah, 295 Chipeta Way, Suite #9, Salt Lake City, UT, 84108, United States, 1 801-213-3599; 2 Biomedical Informatics, University of Utah, Salt Lake City, UT, United States; 3 Pediatrics, Ann & Robert H. Lurie Children’s Hospital of Chicago, Northwestern University Feinberg School of Medicine, Chicago, IL, United States; 4 Utah Data Coordinating Center, University of Utah, Salt Lake City, UT, United States; 5 College of Nursing, University of Utah, Salt Lake City, UT, United States; 6 Intermountain Health, Salt Lake City, UT, United States

**Keywords:** home monitoring, apps, early warning, early intervention, children with medical complexity

## Abstract

**Background:**

Children with medical complexity (CMC) experience frequent acute health deteriorations, leading to recurrent hospitalizations. Home monitoring has been suggested to help reduce hospitalizations among CMC, yet the benefits have not been demonstrated. MyChildCMC is a novel app, co-designed with parents, that supports CMC home monitoring to identify early warning signs of acute health changes to promote timely intervention.

**Objective:**

This study aims to assess the efficacy of the MyChildCMC intervention on child and parent outcomes and the sustainability of MyChildCMC use by parents and of the intervention’s effects.

**Methods:**

This is a 12-month, 2-arm randomized controlled trial recruiting 360 CMC (aged 1‐18 years) and their parents: 180 child-parent dyads assigned to the MyChildCMC app and 180 to the control arm. Participants randomized to the MyChildCMC arm use a sensor and digital thermometer to record vital signs (temperature, heart rate, respiratory rate, and oxygen saturation) and symptoms (pain, feeding/fluid intake, mental status, and seizures) in the app daily and receive immediate feedback. Parents receive alerts for early signs of their child’s health deterioration and can view summary reports and individual graphs about the child’s health status in the app. Alerts are also sent to the care team, who can view, via a web-based dashboard, summary reports and individual graphs to support timely care. Participants randomized to the control arm use the sensor/thermometer to record the same clinical parameters daily in a diary. Both arms receive financial incentives for daily monitoring and for responding to surveys at baseline and at 3, 6, and 12 months. The efficacy will be assessed at 6 months. The monitoring incentive will be stopped at 6 months, but participants will continue with daily monitoring for 6 more months to assess sustainability at 12 months. The primary outcome is the number of hospital admissions among CMC. Secondary outcomes are assessed for the child (hospital days and quality of life) and the parent (stress, self-efficacy, and satisfaction with the child’s care).

**Results:**

We will determine the extent to which the use (vs controls) of the MyChildCMC app leads to reduced emergency department/hospital admissions among CMC. We will also determine whether MyChildCMC use leads to improvements in secondary outcomes. Before the current full-scale study, we conducted a pilot trial, which showed promising results. However, the study was not powered to detect differences in hospital admissions. A larger, definitive study of MyChildCMC’s impact was needed to confirm these results. Recruitment for the current study started on January 6, 2026, and will end by March 31, 2028. To date, 41 participants have been enrolled. We expect the last participant to complete the study by March 31, 2029, and thereafter we will analyze the data.

**Conclusions:**

The MyChildCMC app was designed to support CMC home monitoring and proactive care. If efficacious, it will serve as a new model for reducing hospital admissions, with the potential for broad dissemination.

## Introduction

About 1 in 4 (19 million, or 26.2%) US children younger than 18 years are children with special health care needs [1] and receive health care services beyond those required by typically developing children [1,2]. Among children with special health care needs, there is a subgroup of approximately 3 million of the most vulnerable children, called children with medical complexity (CMC), who receive a high level of specialized medical care involving up to 15 specialists [3]. CMC have complex, multisystem chronic diseases; high health care needs; and frequent acute health deteriorations [2]. Although they represent 6% of the pediatric population, CMC account for 40% of pediatric health care spending and 70% of home health care spending in children [4,5].

The task of clearly defining CMC remains an ongoing challenge for clinicians and researchers alike [5]. A recently proposed, consensus-based, standardized definition of CMC includes specific criteria for diagnostic conditions, functional limitations, health care use, and family needs [6]. Practically, CMC are defined as children who have (1) complex, *multisystem* (≥3 organ systems affected) chronic conditions, due to prematurity, congenital anomalies, genetic syndromes, neuromuscular disorders, and acquired conditions; (2) impairments that result in marked *intellectual/physical disabilities* and functional limitations requiring adaptive equipment (eg, wheelchair); (3) medical technology (eg, mechanical ventilator, tracheostomy tube, cerebrospinal fluid shunt, etc) dependence to sustain life; (4) multiple health care providers involved in their care; and (5) *high medical fragility*, leading to frequent acute health deteriorations and recurrent emergency department (ED) visits and frequent (and often prolonged) hospital admissions [7,8].

In 2022, CMC had 1.02 million hospitalizations, representing 38% of all hospitalizations at freestanding children’s hospitals, 62% at general hospitals, 63% of all pediatric hospitalizations, 93% of inpatient deaths, and 84% of costs (30.6 billion US dollars) incurred by hospitalized children [8]. A recent analysis of national hospitalization data showed that CMC use health care services extensively and have high in-hospital mortality rates compared with non-CMC [9]. Ramgopal et al [10] reported that having medical complexity is associated with almost 10 times the odds of in-hospital mortality. Given the inherent complexity, CMC often do not meet inclusion criteria for clinical studies, including randomized controlled trials (RCTs) and evidence-based interventions [11,12].

Complex care programs are emerging (eg, hospital-to-home transitions, complex care services, medical homes, home health care, telehealth, etc) to support CMC with care coordinators, home care services, community health centers, and/or other complex care services [13-15]. While RCTs are rare, observational studies of these programs report positive results, including reduced frequency and duration of hospitalizations, and decreased costs [16,17]. However, despite early evidence of decreased health care use, CMC continue to experience high rates of hospitalization [13,18]. In 1 study, three-quarters of CMC hospitalizations were deemed avoidable [19]. To date, clinical programs for CMC lack remote monitoring capabilities, which have the potential to improve child and family quality of life (QOL) and reduce the need for hospitalization through early recognition and intervention of acute illnesses [20]. Strategies to reduce the frequency of hospital admissions among CMC are needed [21].

mHealth apps are designed to enable families/caregivers of CMC to enter their child’s vital signs and symptoms and receive real-time feedback to inform just-in-time treatment decisions [7,20,22]. Apps can be used to support symptom monitoring and empower families to recognize and act on early changes in their child’s health status [7,20,22]. MyChildCMC is a new app co-designed with CMC parents to support home monitoring by focusing on common (crosscutting) symptoms (rather than individual diseases) that often precede hospital admissions. In a pilot RCT, use of the MyChildCMC app detected early warning signs of health deterioration in CMC up to 14 days prior to most admissions, allowing time to act to avoid admissions [23]. Compared to controls, app users had fewer hospital days and higher satisfaction [23]. Yet, the pilot trial was not powered to detect changes in the number of hospital admissions. It was limited to parents recording vital signs/symptoms in the app and receiving feedback (*app arm*). The care team was not involved.

The aim of the current study, a fully powered multicenter RCT (with care team involvement to respond and to act on alerts), is to assess MyChildCMC’s efficacy on child and parent outcomes. We also aim to assess the sustainability of MyChildCMC use and intervention effects. We hypothesize that, compared to the control group, participants using the MyChildCMC app with home monitoring will have fewer ED/hospital admissions, fewer hospital days, higher child QOL, higher parent satisfaction, higher parent self-efficacy, and lower parent stress. We also hypothesize that MyChildCMC use and intervention effects achieved at 6 months will be sustained at 12 months.

## Methods

### Study Design

This is a 12-month parallel RCT with 2 arms (MyChildCMC vs control arms) involving CMC (aged 1‐18 years) and their parents (or primary caregivers). Figure 1 presents an overall diagram of the study that complies with the SPIRIT (Standard Protocol Items: Recommendations for Interventional Trials) guidelines (Checklist 1) [24].

**Figure 1.**
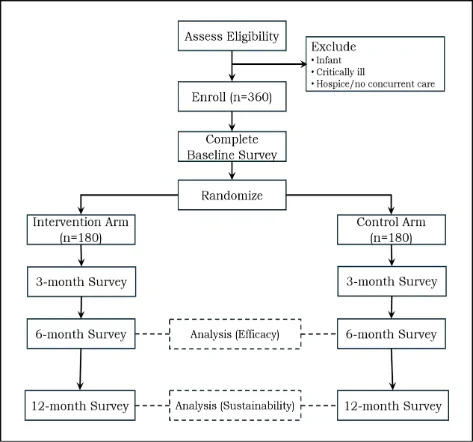
Study diagram.

### Study Participants/Settings

CMC and primary caregivers are enrolled in Salt Lake City (SLC) and Chicago. The SLC site includes the Pediatric Comprehensive Care Program for CMC [25], at the Primary Children’s Hospital (PCH) outpatient clinic, which cares for more than 1000 unique CMC per year. PCH is an affiliated pediatric hospital of the University of Utah Spencer Fox Eccles School of Medicine owned by Intermountain Health. The Chicago site includes the Lurie Children’s Hospital (LCH) Pediatric Care Coordination and Complex Care Programs, which together care for more than 700 unique CMC per year. LCH is an affiliated pediatric hospital of Northwestern University Feinberg School of Medicine.

### Eligibility Criteria

#### Inclusion Criteria

The inclusion criteria are as follows: (1) CMC receiving care at participating sites, (2) CMC’s aged 1 to 18 years, (3) parents willing to use their smartphone (or tablet) and the internet to participate, and (4) parents who speak English or Spanish. CMC are defined as children who meet a combination [(a, b, AND c) or (d AND (a or b or c))] of the following: (a) multisystem chronic diseases involving 3 or more organ systems, (b) 3 or more health care providers involved in their care, (c) high fragility or frequent (≥2) hospitalizations, or 1 prolonged (>10 d) hospitalization in the previous year, and/or (d) medical technology dependence.

#### Exclusion Criteria

CMC are excluded if they are (1) older than 18 years of age, (2) critically ill, (3) an infant, (4) in hospice without concurrent care, or (5) not English or Spanish speakers. Infants are excluded as many CMC younger than 1 year have prematurity-related conditions that may be resolved by at the age of 2 years. CMC younger than 18 years are excluded as some transition to adult care. It should be noted that, to reduce the digital divide among CMC parents, we are providing families without a smartphone or internet with low-cost tablets and cellular internet to allow them to participate. We estimate that approximately 10% of parents of CMC will need this support.

### Recruitment

Participants are identified electronically by the study teams, assessed for eligibility, and invited to participate remotely (by phone or email), during clinic visits, or during hospital admissions. Eligible participants who agree to participate give informed consent and/or parental permission. CMC aged 7 years or older, if cognitively capable, provide assent. CMC who turn 18 years old during the study provide consent or are consented by a legally authorized representative.

### Randomization/Allocation

A computer-generated allocation sequence is built into REDCap for randomization. Randomization is stratified by age group (1‐11 vs 12‐18) and child disability severity (6‐15 vs 16‐30) as these factors may affect child outcomes. Child disability severity is measured at enrollment using the Functional Status Scale (FSS), which assesses the child’s functional status across 6 domains with scores ranging from 6 (normal function) to 30 (very severe dysfunction). Participants are allocated into 2 arms in a 1:1 ratio using a block-randomized scheme with random block sizes of either 4 or 6. Once an eligible participant consents and completes baseline surveys, the research team uses REDCap to identify the allocation number (the actual allocation arm is concealed from the study teams) and then discloses it to the participant.

### Intervention Arm or Experimental Group

The intervention includes MyChildCMC, an app designed to support remote monitoring to help identify early acute changes in CMC’s health status to promote timely care [26]. The app is available in the Apple App Store and Google Play Store, but access is currently limited to study participants. Parents in the intervention arm receive the app, a sensor, and a thermometer, and training on the study and on measuring and recording vital signs and symptoms in the app. The intervention group also receives help to download the app from the Apple App Store or Google Play Store, an authorization code to access the app, and help in setting up a Bluetooth connection with the sensor to allow the automated transmission of vital signs data. This group uses the sensor/app daily for 12 months postenrollment to monitor their child’s vital signs/symptoms and receive reminders and real-time feedback (summary reports and individual graphs), as well as alerts from the app for abnormal changes in vital signs and symptoms. Alerts indicating early warning signs of deteriorating health in the child with CMC are sent to the parent and care team for follow-up. The care team can view patient data and individual graphs via the dashboard. The app includes the following features [26]:

1. Vital sign sensor: A Food and Drug Administration (FDA)–approved pulse oximeter [27] is briefly attached to the child once daily to record not only oxygen saturation (O_2_sat) but also heart rate (HR) and respiratory rate (RR) to monitor cardiorespiratory stress and oxygenation. A Bluetooth connection is established to allow automatic data transmission between the sensor and the MyChildCMC app on smartphones and tablets.
2. User interface: The app uses touch screens with visually appealing, easy-to-understand pictograms, radio buttons, and color-coded graphs, making it user-friendly for individuals with low technological literacy. It allows daily recording of temperature (Temp) to monitor fever and symptoms (pain, fluid intake/feeding, mental status, and seizures) not captured by the sensor. For pain, the Numeric Rating Scale (NRS) is used [28], which is a 1-item parent proxy scale categorizing pain as mild (0‐3), moderate (4-7), or severe (8-10). Food/fluid (1-item) is recorded as 25%, 50%, 75%, and 100% of usual intake. Mental status, using the behavior (1-item) question of the Pediatric Early Warning Scores (PEWS), is scored as follows: (1) playing/appropriate, (2) sleepy, (3) irritable/agitated, and (4) lethargic/unresponsive. Seizures are recorded as (1) fewer than usual, (2) usual, (3) more than usual, and (4) way more than usual (or not applicable, for children who do not usually have seizures).
3. Automated reminders: Daily reminders via text message and/or email (depending on user’s choice) are sent to users to support adherence to daily use.
4. Decision rules for alerts: MyChildCMC uses abnormal thresholds and run chart rules [29,30] to detect significant changes in vital signs/symptoms. For thresholds, abnormal values are as follows: O_2_Sat of less than 90%, pain score of more than 7, temperature of less than 96.8° or more than 101.3 °F, less than 75% or 25% or above in hydration/nutrition, and scores of 3‐4 for mental status. CMC often have baseline temperature, HR, RR, pain, and O_2_Sat values outside the published ranges for typical children when they are not sick. Thus, run chart rules are used to detect significant changes from the *patient’s own baseline*, defined as *outliers* (3 consecutive data points 2 SDs away from the patient’s baseline in a week), a *shift* (≥7 consecutive data points below/above the mean), or a *trend* (≥7 consecutive increasing/decreasing data points). Patient baselines are established with 14 or more days [29,30] of data when the child’s health status that day is “typical” (per the parent). Typical day data are used to calculate the patient’s average and SD. Subsequent data are then compared to the patient’s own baseline to detect significant changes (Table 1).
5. Real-time feedback: It includes a summary report and individual graphs, displaying changes in vital signs and symptoms in 2‐3 colors: green (no outlier, trend, or shift), yellow (trend or shift), or red (outlier, shift, or trend). Each color is linked to specific feedback (green=“good,” yellow=“follow closely,” red=“not good”) and automatic alerts for parents/care teams if a value is in Yellow one or more consecutive days or Red Zone.
6. Automated alerts: Text or email alerts are sent *to the parent* when abnormal thresholds or run chart rules are met. Alerts are also sent *to the child’s care team*, asking them to log in to the dashboard within 24 hours to view the affected patient’s data (summary/individual graphs), assess, and address their needs.
7. Motivational feature: MyChildCMC has a *progress bar* that adds points for completion of a daily assessment. At 100 points (~2 wk), a congratulatory message is generated, and the progress bar then resets to 0. The progress bar is used to promote consistent app use.
8. Photo function: The app allows the inclusion of a child’s photo in the patient profile for immediate visual recognition by the care team, assisting in patient care. Also, the app allows the inclusion of *current photos* to help the care team assess a child’s health issue. These photos are accessible via the dashboard (see below).
9. Comment (free text) field: A comment box helps parents communicate any important issues to the care team and to record interventions attempted at home (if they wish) when a child’s parameter is abnormal.
10. Medication list function: This optional function allows parents to record and store medication names, prescribed doses, and frequencies.
11. The dashboard: A web interface allows the care team to monitor the CMC population remotely. It provides real-time access to app data. Sorting and filtering menus allow users to drill-down to a group (eg, those doing well [green cells], not doing well [yellow/red cells], and not adherent with daily use [empty cells]), or access individual patient data, graphs, comments, and the child’s photos, to promote proactive patient care. When there is an alert, the patient is automatically flagged (indicating that an issue needs to be identified and resolved) in the dashboard until a care team member completes a checklist that ensures core elements of the protocol are delivered with fidelity, including whether the parent was contacted, whether an action was taken, and whether the issue was resolved, and then the record is unflagged when all options are checked (all actions completed). Over the weekend, alerts are sent to on-call physicians. The care team can also view users who are nonadherent with daily use so the team can reach out to families to assess and resolve the reasons for nonadherence.
12. Server: MyChildCMC data are stored behind the University’s firewall on a Health Insurance Portability and Accountability Act (HIPAA)–compliant server.

**Table 1.** Parameters, sources, and decision support rules in MyChildCMC.

Parameter	Source	Rule type	Alert decision rule
Temperature	Sensor	Run chart/threshold	Shift or >2 SD or temperature >96.8 or >101.3
Heart rate	Sensor	Run chart	Shift or >2 SD
Respiratory rate	Sensor	Run chart	Shift or >2 SD
Oxygen saturation	Sensor	Threshold/run chart	<90% or shift or >2 SD
Pain score	Parent	Threshold/run chart	>7 or shift or >2 SD
Feeding/hydration	Parent	Threshold	<75% hydration or ≤25% nutrition
Seizures	Parent	Threshold	Score 3 or 4
Mental status	Parent	Threshold	Score 3 or 4

### Control Arm

Participants randomized to the control arm receive a sensor, a thermometer, a logbook (paper diary), and training by the research team on measuring and recording the child’s vital signs/symptoms. Participants in the control arm use the sensor and diary daily for 12 months postenrollment to record and monitor the same vital signs/symptoms as the intervention group, but they do not receive reminders, feedback, or alerts. They are instructed to follow up with the child’s care team on their own as usual if they have any concerns about the child’s health.

### Usual Care

Both arms receive their regular clinical care from their care teams, which includes (1) a shared care plan; (2) education about potential interventions to try at home if a child’s parameter is abnormal; (3) a social worker’s consultation and connection to community resources; (4) coordination of medical care, referrals, and appointments, in collaboration with health care providers; and (5) inpatient consultation for hospitalized CMC.

### Incentives

Participants in both arms receive monitoring financial incentives for daily use of the app (intervention arm) or diary (control arm) for the first 6 months of the study, totaling US $120 (~US $10 every 2 weeks for 6 months). App usage (adherence percentage) for every 2 weeks is automatically recorded and made accessible via the dashboard. Diary usage (for every 2 weeks) is monitored through photos of completed logbook pages uploaded by study participants that are accessible through REDCap for research team verification. Participants with greater than 50% adherence to either the app or diary for each 2-week period are sent a US $10 gift card. The efficacy of the MyChildCMC intervention will be assessed at 6 months. We will stop providing monitoring incentives at the end of 6 months and follow participants for 6 more months to assess sustainability at 12 months. All participants also receive US $50 for each survey completed at enrollment and at 3, 6, and 12 months (US $200 total for responding to surveys). Overall, participants receive US $320 total if they complete all study activities.

### Outcome Measures

Efficacy will be assessed at 6 months postenrollment, by comparing multiple outcomes for the child (ED/hospital admissions, number of hospital days, and QOL) and parent (satisfaction, self-efficacy, and stress). The primary outcome measure is the number of ED/hospital admissions (6 months preenrollment and 6 months postenrollment), which is an important public health concern, as CMC have recurrent hospitalizations that account for 40% of all hospital admissions and one-third of health care costs in US children [31]. Secondary outcome measures are total hospital days (6 months preenrollment and 6 months postenrollment), change from baseline in the child’s QOL, parent stress, parent self-efficacy, and parent satisfaction with overall childcare. Sustainability will be assessed at 12 months by comparing adherence and intervention effects between the first and second 6-month periods.

### Data Collection

#### Efficacy

ED/hospital admissions and hospital days (6 months pre/postenrollment [for impact analysis] and 7‐12 months postenrollment [for sustainability]) will be collected electronically using the Intermountain Enterprise Data Warehouse (EDW) [32], an integrated database that links data from 22 hospitals and 180 clinics in Utah, capturing health care services for 95% of CMC in Utah. ED/hospital admissions and hospital days will also be collected from the LCH EDW, which captures 70% to 80% of CMC health services in Chicago. To supplement the LCH EDW’s data, we will use the Chicago Area Patient-Centered Outcomes Research Network (CAPriCORN) data [33], which stores data from 11 health care systems/hospitals in Chicago, and will help capture most of the use data occurring outside LCH systems. *Child’s QOL* will be collected using a CMC-specific survey validated by Ellzey et al [34]. The 7-item survey assesses physical health, mental health, sleep, pain, activities, and general QOL. *Parent Stress Level* will be assessed using the 12-item Zarit Burden Interview (ZBI-12) [35], a self-administered validated survey with 5 response options ranging from 0 (never) to 4 (always). It assesses stress/coping, negative affect, and social support, with the total score rating caregiving stress (burden) as no/little (0‐2), mild-to-moderate (3-7), moderate-to-severe (8-18), and high/severe (>18). *Parent Self-Efficacy* will be collected using a CMC-specific questionnaire adapted from validated surveys, including the PROMIS and PhenX Toolkit self-efficacy scales, with adaptation needed because no existing scale meets CMC needs [36]. *Parent Satisfaction with Child’s Care* will be collected using questions adapted from the Client Satisfaction Questionnaire [37]. Both arms will complete surveys (including child’s QOL, parent caregiving self-efficacy, and stress) at baseline and 3, 6, and 12 months (Multimedia Appendix 1) and parent satisfaction (baseline and 6 months). The survey name, target, instrument, and timeline are in Table 2 (Spanish versions are available).

**Table 2.** Data collection, instruments, and timelines.

Study variable	Target	Instrument	Baseline	3 months	6 months	12 months
Demographics	Caregiver	N/A	✓			
Disability severity	Child	Functional Status Scale	✓			
Social support	Caregiver	OSSS-3^a^	✓			
QOL^b^	Child	Ellzey CMC^c^ QOL Questionnaire	✓	✓	✓	✓
Health literacy	Caregiver	Brief Health Literacy Screening Tool	✓			
Stress	Caregiver	ZBI-12^d^	✓	✓	✓	✓
Self-efficacy	Caregiver	Self-Efficacy Scale	✓	✓	✓	✓
Satisfaction	Caregiver	Parent Satisfaction Questionnaire	✓		✓	

a OSSS-3: 3-item Oslo Social Support Scale.

b QOL: quality of life.

c CMC: children with medical complexity.

d ZBI-12: 12-item Zarit Burden Interview.

#### Sustainability

MyChildCMC use adherence will be assessed separately for the first (months 1‐6) and second (months 7‐12) 6-month periods postinitiation and will be measured as the number of logins with complete vital sign/symptom recordings over these periods. Sustainability, the extent to which MyChildCMC use (logins and completed recordings) is sustained will be assessed as follows: a financial incentive related to MyChildCMC use will be provided until the end of the first 6 months. For the second 6 months, users will continue to receive automated reminders, feedback, alerts, and so forth (all app functionalities) but not the monitoring incentive (the incentive for daily use of the diary will also be stopped in controls). We will assess use adherence at 6 months and sustainability at 12 months.

### Statistical Analysis

#### Efficacy

Analyses will be based on intention-to-treat according to randomized assignment, irrespective of adherence. We will use descriptive statistics with means/SDs or medians/IQRs for continuous variables, and frequencies/percentages for categorical variables to compare baseline characteristics between the study arms. We will compare the rate of ED/hospital admissions at 6 months postenrollment between the 2 arms using Poisson or negative binomial regression with dispersion, possibly augmented to account for 0 inflation if the proportion of participants with 0 ED events or hospitalizations exceeds that accounted for by the model [38]. We will also compare child QOL total score between the 2 arms at baseline and at 3 and 6 months, using a linear mixed-effect model (LMM), with a random intercept to account for correlation among repeated measurements within the same patient. Adjustment for baseline QOL total score will account for any baseline imbalances in the outcome and regression to the mean [39]. Also, secondary contrasts will estimate the treatment effect on the mean total score at each follow-up visit and compare treatment effect sizes between different follow-up visits to assess whether the treatment effect attenuates or increases over time. Furthermore, we will compare other secondary outcomes (hospital days, parental stress, self-efficacy, and satisfaction) between the 2 arms using LMMs for numeric outcomes.

#### Sustainability

For MyChildCMC use adherence, we will determine the number of logins with complete vital sign/symptom recordings (out of all opportunities) over the first 6 months (months 1‐6, or period 1) and the second 6 months (months 7‐12 or period 2) at the individual parent-level and categorize parents into *high* (>50% logins) and *low* (≤50% logins) users (*see Aim 2*). For sustainability, we will compare parent-level login performance between period 1 and period 2 and categorize parents as *sustainer* (if MyChildCMC use adherence at 12 months, or at the end of period 2, remains at or above the level achieved at the end of period 1), *otherwise nonsustainer*. We will also assess sustainability of the intervention effect by comparing outcomes (ED/hospital admissions, total hospital days, QOL scores, parent stress scores, parent self-efficacy scores) between period 1 and period 2.

#### Moderator Analysis (or Confounding)

Covariates include parents’ sociodemographics (age, race/ethnicity, marital status, education, household income), social support score, child’s age, insurance (commercial vs public), total number of caregivers, adherence to MyChildCMC use, and, in secondary analyses, the child’s disability severity score. In addition, the possibility that the intervention effect may differ will be evaluated based on the child’s and caregiver’s/parent’s sex as a biological variable, and the child’s age groups (1‐12 and 12‐18). It should be noted that the child’s age and disability severity are among the part of covariates and are used as potential effect moderators in aims 1‐3, as child and parent outcomes can be influenced by the child’s disability severity and the duration of impairment (with parents gaining experience over time).

#### Analytic Design Considerations

Differences in participants’ demographics between sites may affect the generalizability of the study results. Therefore, we will include site as a random effect in the analyses for aims 2 and 3 to adjust for between-site differences. Finally, because sites are referral centers for CMC from surrounding states, the results of this study can be generalized to CMC in multiple states.

#### Retention Strategies and Missing Data

We will use multiple strategies to support retention, including providing financial incentives, mailing thank-you and birthday cards, and confirming contact information at each follow-up survey. Yet, some loss to follow-up is inevitable even with optimal strategies. All analyses will use available data for all participants, irrespective of adherence to the intervention. For LMMs, participants who missed measures (primary and/or secondary outcomes) at certain but not all assessment time points will be included in the analyses, which will be performed using restricted maximum likelihood estimation and will therefore remain approximately unbiased in the presence of missing data as long as the pattern of missingness is random. If the proportion of missing data is greater than 20%, we will use inverse probability of censoring weights or multiple imputation (with mixed-effects pattern-mixture models) to limit the effects of missing data [40-42]. There may still be missingness in the outcome (ED/hospitalizations) due to limitations of the data warehouses used, although it is likely a small percentage based on preliminary data. We are combining site-specific and regional data (All Payer/Utah; CAPriCORN/Chicago), helping us to capture essentially all use data.

#### Sample Size Calculation

Our pilot RCT showed a pooled group baseline rate of 0.98 per 3 person-months for the primary outcome (ED/hospital admissions) or a background rate of 1.96 per 6 person-months of our target follow-up period. Thus, we plan to enroll 360 child-caregiver dyads with an estimated 15% to 20% loss to follow-up, leading to 288 to 306 participants completing the study. Assuming an average pre-post correlation of at least 0.5 on the primary outcome which reduces the sample size by 1.5 as a design effect, we will have equivalent sample sizes of 384 to 408 participants over the 2 time periods. A Poisson regression of ED/hospital admissions with a sample of 384 participants (20% loss to follow-up) achieves 80% power to detect a response rate ratio of 0.744 or less for the intervention vs control arms, at the 0.05 significance level. If there are other standardized variables being adjusted in the model, and a multiple regression of the control/intervention arms on other adjusted covariates is expected to have an *R*^2^ of 0.20, the detectable rate ratio will be 0.717. With 408 total participants completing the study (15% loss to follow-up), the detectable rate ratio will be 0.751, and if there are other standardized variables being adjusted in the model with an *R*^2^ of 0.20, the detectable rate ratio will be 0.725.

### Ethical Considerations

The study was approved by the University of Utah Institutional Review Board (IRB; IRB approval/reference number 00179595) which is also acting as the Single IRB for both the Utah and Chicago sites. Study participants provide consent before completing any study procedures. During the consenting process, participants are informed about the voluntary nature of the study that they can withdraw at any time without consequences and that precautions are in place to ensure the confidentiality of the data and the privacy of study participants. All data and other information collected during the study are stored on a University of Utah HIPAA-compliant secured server, accessible only to the research team. As this is an RCT, the study was registered on ClinicalTrials.gov (ID: NCT06883045).

## Results

We will determine the extent to which use (vs nonuse) of the MyChildCMC app leads to reduced ED/hospital admissions in CMC. Before the current full-scale study, we conducted a pilot trial in which a total of 62 parent/CMC dyads were invited to participate: 50 parent/CMC dyads were enrolled (80.6% enrollment rate). Of the 50 parents/CMC, 24 were randomized to the MyChildCMC arm and 26 to the control arm. Participants were enrolled between June 10, 2019, and December 19, 2019, and asked to monitor their child’s vital signs/symptoms daily for 3 months after hospital discharge, using the MyChildCMC app (intervention arm) or paper (control arm). There was no other interaction or interference with the participant’s usual care. The retention rate was 80% (40/50) at 1 month and 76% (38/50) at 3 months. The engagement rate was 68.3% at 1 month and 62.6% at 3 months. Run chart shifts in vital signs were a common finding preceding 9087% (13/15) of admissions. The satisfaction score was 26.9 in the MyChildCMC group and 24.1 in the control group (*P*=.04). QOL or subsequent admissions did not differ between the groups. The 3-month hospital days (pre-post enrollment) decreased from 9.25 to 4.54 (rate ratio: 0.49; 95% CI 0.39‐0.62; *P*<.001) in the MyChildCMC group and increased from 1.08 to 2.46 days (rate ratio: 2.29; 95% CI 1.47‐3.56; *P*<.001) in the control group. We concluded that the trial was feasible and that MyChildCMC appeared effective in reducing hospital days and showed the ability to detect early warning signs (mostly as shifts in vital signs) of health deterioration in CMC to promote timely interventions that might help avoid hospital admissions. However, the pilot trial was not powered to detect differences in hospital admissions. The current study is a larger, definitive study designed to confirm these results, which is the goal of the current study. Participant recruitment for the current study started on January 6, 2026. To date, 41 participants (parent/CMC dyads) have been enrolled. We expect participant enrollment to end by March 31, 2028, and the last participant to complete study activities by March 31, 2029, after which we will start analyzing the data to publish our results by October 30, 2029.

## Discussion

### Principal Findings

Compared to the control arm, we expect the current study to show that MyChildCMC users have fewer hospital admissions, fewer hospital days, lower parental stress, and higher child’s QOL scores, parent satisfaction, and parent self-efficacy in managing their child’s health. The expectation of positive results is based on MyChildCMC's ability to MyChildCMC to help detect signs of health deterioration in CMC early (up to 14 days in advance) and to support timely care, which can help reduce hospital admissions. Frequent hospital admissions among CMC can lead to poorer QOL. Also, long-term caregiving can be stressful and lead to poor caregiver’s health [24,43-46]. Thus, a reduction in hospital admissions may lead to improved child QOL, reduced caregiver stress, and improved caregiver satisfaction. We also expect that adherence to MyChildCMC and intervention effects achieved at 6 months will be sustained at 12 months as the app may offer tangible benefits and value to parents of CMC (eg, reduction in hospital visits).

Improved survival of children with conditions previously incompatible with life have led to growing CMC populations [47-51]. Also, a recent paper has shown that CMC hospitalizations have increased over the past decade [8]. Due to high medical fragility, CMC experience frequent acute health deteriorations, leading to recurrent hospitalizations and high health care costs [11,52-58]. Despite high costs, CMC are often excluded from clinical studies including RCTs, and evidence-based interventions are rare [11,59]. To reduce ED/hospital admissions for CMC, remote monitoring has been suggested [60-62]. For children, this enables parents, in collaboration with a care team, to better manage their child’s condition at home by monitoring symptoms and/or behaviors, identifying/responding to early signs of deterioration, and seeking/providing timely care when needed.

There is traction for using apps to support remote monitoring, improve care, and reduce CMC hospitalizations [7,20,60-62]. Effective remote monitoring of chronic diseases can enhance care coordination, improve outcomes, and reduce admissions [7,20,63-72], mainly due to early recognition and timely management of acute changes in a patient’s health status [7,20,31,50,73-78]. mHealth apps are generally used to support remote monitoring [61,62] of single chronic diseases (eg, asthma, diabetes, etc) [79-84] and empower families and care teams to recognize and act on early changes in a patient’s health status [7,20,60,85]. However, no such apps have been implemented for CMC. To date, few authors have reported on the usability of a remote monitoring app, but these apps target only a subset of CMC (eg, those with enteral feeding tubes), with no evaluation of impact [7,20,22,86]. Also, Werner et al [87] conducted an RCT to assess the impact of an app designed to improve medication administration accuracy but not the health status of CMC. Variable, multisystem conditions make it difficult to develop remote monitoring apps for CMC. Despite multiple and variable underlying conditions, hospital admissions for CMC are often preceded by a limited set of shared or common (crosscutting) acute symptoms, including fever, respiratory distress with decreased oxygenation, feeding/fluid intolerance, altered mental status (sometimes seizures), and pain [62,88,89]. MyChildCMC was developed to monitor and identify early changes in cross-cutting symptoms among CMC.

Our study is unique as it introduces a novel and practical approach to monitoring all CMC by targeting crosscutting symptoms, rather than individual conditions or isolated symptomatic complaints (ie, respiratory vs neurologic), making remote monitoring for CMC possible despite multiple and variable conditions. The MyChildCMC intervention is also aligned with the Institute of Medicine, which warned against designing care for patients with multiple chronic diseases around single-disease interventions rather than around common features [90,91]. Also, the use of an FDA-approved sensor, integrated with the MyChildCMC app (via Bluetooth), allows automated and accurate recording of vital signs data in the app, minimizing the data-collection burden on families. Long-term caregiving can negatively impact the health of parents of CMC. Our study will be the first to explore if remote monitoring not only improves CMC outcomes but also improves parents’ caregiving self-efficacy in managing child’s conditions and reduces parental stress. Finally, CMC are often excluded from clinical studies, and RCTs involving CMC are rare [11,59]. Our study is unique in that it is an RCT involving CMC.

Our study protocol has a few potential limitations that may affect the interpretation of the results. First, one may think that our study population’s age range (1‐18 y) is too wide. This age range was included because CMC exhibit similar developmental and functional abilities and typically function below what is expected for a typical 7-year-old child, allowing the inclusion of a wide age range in research studies. Second, one may think that future dissemination of MyChildCMC intervention will be challenging in terms of sensor costs, manpower, and time for training. Further, the time for care coordinator and clinician review is variably reimbursed across Medicaid programs and private pay plans, which may limit sustainability. However, if MyChildCMC is found to be associated with a significant reduction in hospital admissions, the results could be used as an important evidence base to justify its medical necessity and potential cost savings to the health system, as well as an incentive for health care insurances to support sensor-related costs. Also, after initial and one-time training (that can be provided by care coordinators), use of sensor/app is straightforward, involving only parents/caregivers. Follow-up care triggered by MyChildCMC use is standard of care for CMC. We are anticipating our future cost-effectiveness study to demonstrate significant cost savings as hospital admissions account for most CMC-related health care costs. Third, because of the type of our intervention, it was not possible to blind study participants to the intervention (MyChildCMC) or control (diary). Therefore, the intervention group may report better outcomes as they may be more motivated to participate than the control group, leading to potential bias. However, the primary outcome (hospital admissions) will be collected electronically. This will minimize the risk bias in the reporting of the intervention effect. Finally, we are providing similar study materials (sensor and digital thermometer) and incentives (for daily monitoring and responding to surveys) to both study arms. The difference is that the intervention group monitors their child’s vital signs/symptoms using the app, while the control group monitors the same parameters using a diary. Therefore, the results obtained from the study will reflect the true impact of MyChildCMC app.

### Conclusion and Future Direction

MyChildCMC is a promising app that has been shown in a pilot trial to improve CMC and parent outcomes. If efficacious, MyChildCMC will serve as a new model for improving care and reducing hospital admissions in CMC, which can be generalized to all children with multiple chronic conditions, with potential for national dissemination to reduce CMC-related care burden in the United States.

## Supplementary material

10.2196/108485 Multimedia Appendix 1 Survey instruments.

10.2196/108485 Checklist 1 SPIRIT 2025 checklist.

10.2196/108485 Peer Review Report 1 Peer review report by the National Institutes of Health.

## Abbreviations

CAPriCORN: Chicago Area Patient-Centered Outcomes Research Network
ED: emergency department
EDW: Enterprise Data Warehouse
FDA: Food and Drug Administration
FSS: Functional Status Scale
HIPAA: Health Insurance Portability and Accountability Act
HR: heart rate
IRB: Institutional Review Board
LCH: Lurie Children’s Hospital
LMM: linear mixed-effect model
NRS: Numeric Rating Scale
O_2_sat: oxygen saturation
PCH: Primary Children’s Hospital
PEWS: Pediatric Early Warning Scores
QOL: quality of life
RCT: randomized controlled trial
RR: respiratory rate
SLC: Salt Lake City
SPIRIT: Standard Protocol Items: Recommendations for Interventional Trials
ZBI-12: 12-item Zarit Burden Interview
