# Supplementary material for: The Efficacy of a Home Monitoring App for Children With Medical Complexity: Protocol for a Randomized Controlled Trial
Source: JMIR Res Protoc. 2026 Oct 22;15:e108485. doi: 10.2196/108485 (PMC13717321; doi:10.2196/108485)
Supplement: Supplementary file 1 [file resprot-v15-e108485-s003.pdf]

SUMMARY STATEMENT
(Privileged Communication)

PROGRAM CONTACT:
Karen Huss
301.594.5970
hussk@mail.nih.gov

Release Date: 08/03/2023
Revised Date:

Application Number: 1R01NR020784-01A1
Formerly: 1R01NR020784-01

Principal Investigator

NKOY, FLORY L

Applicant Organization: UNIVERSITY OF UTAH

Review Group: ZRG1 HSS-B (90)
Center for Scientific Review Special Emphasis Panel
Topics in Health Services Research

Meeting Date: 07/11/2023
Council: OCT 2023
Requested Start: 09/01/2023
Opportunity Number: PAR-20-180
PCC: ACGKH
Dual IC(s): MD

Project Title: A Novel mHealth Intervention to Improve Outcomes of Children with Medical Complexity
SRG Action: Impact Score:30 Percentile:14 #
Next Steps: Visit https://grants.nih.gov/grants/next_steps.htm
Human Subjects: 48-At time of award, restrictions will apply
Animal Subjects: 10-No live vertebrate animals involved for competing appl.
Gender: 1A-Both genders, scientifically acceptable
Minority: 1A-Minorities and non-minorities, scientifically acceptable
Age: 6A-Children and Adults, scientifically acceptable

Project Year	Direct Costs Requested	Estimated Total Cost
1	435,566	677,136
2	437,533	680,194
3	441,461	686,300
4	442,580	688,040
5	447,055	694,997
TOTAL	2,204,195	3,426,667

ADMINISTRATIVE BUDGET NOTE: The budget shown is the requested budget and has not been adjusted to reflect any recommendations made by reviewers. If an award is planned, the costs will be calculated by Institute grants management staff based on the recommendations outlined below in the COMMITTEE BUDGET RECOMMENDATIONS section.
ADMINISTRATIVE NOTE
BUDGET MODIFICATIONS
NEW INVESTIGATOR

1R01NR020784-01A1 Nkoy, Flory

COMMITTEE BUDGET RECOMMENDATIONS
NEW INVESTIGATOR
PROTECTION OF HUMAN SUBJECTS UNACCEPTABLE
SCIENTIFIC REVIEW OFFICER'S NOTES

RESUME AND SUMMARY OF DISCUSSION: The proposed research will assess the efficacy and sustainability of MyChildCMC, a mobile health application (mHealth app) designed to monitor and identify early signs of crosscutting symptoms in children with medical complexity (CMC), in a 2-arm randomized controlled trial with CMC (age 1-18 years) and their parents. While the panel acknowledged the high significance of a technology-based home monitoring system to reduce CMC's emergency department and hospital use, reviewers expressed concern that the application did not adequately address the rigor of the prior research. The panel was confident that the Aims of the study could be achieved given the composition of the investigative team. The project was considered highly innovative in its focus on remote monitoring of cross-cutting symptoms in an understudied population. The committee believed this resubmission to be highly responsive to the concerns raised in the prior review. The panel identified several strengths of the application during the discussion, including a strong recruitment strategy, robust quantitative analytic plans, and an app that is currently available in Spanish. The panel also noted some weaknesses with the approach, including concerns that important stakeholders are not included in the advisory committee and that the qualitative approach is under-developed; some reviewers considered it a major weakness that the technology development did not include an equity focus. Following discussion, the difference of opinion among reviewers regarding the approach was unresolved, but a majority of the committee remained enthusiastic about this application that is likely to have a high impact on the field of pediatric medicine.

DESCRIPTION (provided by applicant): Children with medical complexity (CMC) are the most vulnerable of children with chronic diseases, who have complex, multisystem chronic diseases affecting ≥3 organ systems, severe functional limitations and technology dependencies. CMC have high health care needs, and account for 40% of hospitalized children and 35% of all pediatric health care costs. Due to high medical fragility, CMC have frequent acute deteriorations superimposed on their chronic conditions, leading to recurrent emergency department (ED)/hospital admissions and affecting quality of life (QOL). To reduce ED/hospital admissions for CMC, remote monitoring is suggested, with use of mHealth apps to regularly assess their health status remotely and identify early signs of acute deterioration, allowing for early interventions to prevent ED/hospital admissions. Yet no app to support remote monitoring of CMC exists. Variable, multisystem conditions among CMC make it difficult to develop an app. Also, many CMC are at high-risk for health care inequities, with minorities having higher unmet needs, but the impacts of health care inequities and social determinants of health (SDOH) on ED/hospital admissions in CMC are rarely studied. Fortunately, ED/hospital admissions for CMC are often preceded by a limited set of shared (crosscutting) acute symptoms. These crosscutting symptoms rarely occur suddenly. Studies suggest that they usually start as subtle signs, often unnoticed by parents until they escalate to prompt an ED/hospital visit. Thus, crosscutting symptoms offer an opportunity for a novel and practical approach for developing a remote monitoring app for CMC, despite their multiple, variable underlying conditions. In a focus group, parents identified the crosscutting symptoms that most often preceded their children's hospital admissions, and conveyed their needs, preferences and key functionalities that led to MyChildCMC, the first app designed to monitor and identify early signs of crosscutting symptoms in CMC. In a pilot trial of 50 subjects, we confirmed feasibility of MyChildCMC use by parents, ability to detect early signs 2-14 days prior to ED/hospital admissions, and use leading significantly to fewer hospital days than controls. The current study will assess the efficacy and sustainability of MyChildCMC in a fully-powered 6-month, 2-arm (MyChildCMC

vs usual care) trial of CMC (age 1-18 years) and their parents. Parents assigned to MyChildCMC will use the app daily for 6 months, both arms will receive financial incentive for participation, then we will stop the incentive and follow subjects for 6 more months to assess sustainability at 12 months. We will also assess if MyChildCMC use will help reduce or eliminate inequities in ED/hospital admissions. Specific Aims are: 1) Determine MyChildCMC's efficacy on 1.a. Child (ED/hospital use, hospital days and QOL) and 1.b. Parent (satisfaction, self-efficacy and stress) outcomes; 2) Compare ED/hospital use among racial/ethnic and SDOH subgroups to assess potential effect of MyChildCMC on inequities; 3) Use mixed methods to identify child and parent factors associated with MyChildCMC's use adherence and sustainability.

PUBLIC HEALTH RELEVANCE: Findings from this project will provide an effective, practical, and generalizable approach to supporting home monitoring of children with medical complexity (CMC). This will also serve as a model for reducing recurring emergency department (ED) visits and hospital admissions associated with CMC, generalizable to all children with other multiple chronic conditions.

CRITIQUE 1

Significance: 2
Investigator(s): 2
Innovation: 2
Approach: 3
Environment: 1

Overall Impact: Despite substantial needs of children with medical complexity (CMC) that result in frequent and recurrent hospitalizations and related substantial costs, no remote monitoring app exists for this population that also accounts for variable, multisystem conditions. This resubmission of an R01 from a New Investigator seeks to test a technology-based remote monitoring app with children with medical complexity and examine its efficacy using a multicenter randomized trial design in Utah and Chicago, impact on reducing inequities among racial/ethnic and social determinants of health subgroups, as well as association of child and parent factors with adherence in use and sustainability after the initial 6-month trial period over the next 6-month period. This study has numerous strengths, including fulfilling a major gap in caring for children with medical complexity. The rationale is quite strong. The investigative team is strong, collaborative, and has the expertise and research environment needed to successfully complete the aims. Innovation is very strong given few interventions ever having been conducted with this population let alone interventions using technology. Risk of contamination addressed by assigning different care coordinators to usual care, but not addressed at the clinician level given participants at both sites will be randomized to each arm. Other areas that could benefit from greater description include: conceptual framework for proposal that addresses mechanistic approach for study outcomes, how fidelity monitoring of app will occur, whether parents who are minors themselves will be eligible for study recruitment who have children with medical complexity and associated human subject protections for this more vulnerable population (and their children), and need to include steps for providing on-call clinician backup for care coordinators at each site as part of human subjects protocol. Clarification is also needed regarding qualitative portion of Aim 3. The overall strengths of the approach outweigh these minor weaknesses; this is a very interesting and important application for improving clinical practice.

1. Significance:

Strengths

- Proposal addresses a population – children with medical complexity – who are often excluded from clinical studies including randomized controlled trials (RCTs) and evidence-based interventions.
- Proposal will extend scientific knowledge by using technology and remote monitoring with children with medical complexity.
- Proposal aims to change treatment approach via its approach with technology-based remote monitoring with care coordination to detect early, subtle vital signs that are often unnoticed or overlooked by parents before escalation and that even if noticed, could assist parents who otherwise may struggle with interpretation or deciding a course of action.

Weaknesses

- Strengths and, particularly, weaknesses of rigor of prior research not explicitly articulated.

2. Investigator(s):

Strengths

- High caliber and experienced team with history of collaboration.
- Team has expertise in study coordination and data management.
- Investigators include specific experience expertise analyzing clinical trials with advanced biostatistics.

Weaknesses

- None noted by reviewer.

3. Innovation:

Strengths

- This RCT will be one of the first to focus on children with medical complexity and use of an mHealth app to support health remote monitoring that integrates use of an FDA approved wearable sensor to examine both child and parent outcomes that if found to be efficacious can potentially shift clinical practice.
- This RCT will aim to reduce health disparities among populations of children with medical complexity experiencing disparities.
- This proposal will examine factors that influence user engagement as well as sustainability of use after the intervention that will help refine future improvements of the app.

Weaknesses

- Cost effectiveness analysis is not included as part of current proposal.

4. Approach:

Strengths

- Multicenter study designed to test *MyChildCMC's* efficacy in a 6-month trial of children aged 1-18 years (and parents) on children's ED/hospital use, hospital days, and quality of life (QOL) as well as parents' satisfaction, self-efficacy, and stress outcomes.
- RCT designed to also reduce or eliminate inequities as a result of its use.

- Examining child and parent factors associated with technology's use adherence and sustainability using quantitative and qualitative data approaches.
- Use of randomization approach by age and disability status.

Weaknesses

- (Minor) Self-monitoring framework and health behavior change theories described as study frameworks; but mechanistic approach for study outcomes not well described.
- (Moderate) Contamination risk given same clinicians will be interfacing with all randomized trial participants.
- (Minor) Children under 18 and parents will be recruited for study with parent permission/consent and minor assent (as possible); consideration may need to be made for parents who are minors who may represent more vulnerable populations and require additional protections.
- (Minor) Mixed methods framework to analyze Aim 3 mixed methods study approach not well-described.
- (Moderate) Unclear approach for fidelity monitoring of app.
- (Minor) Steps for providing on-call clinician backup for care coordinators at each site.
- (Minor) Creating "authenticated links" with Cerner/EPIC EHRs is mentioned but never described – it is not clear what this means and barriers to workflow issues (e.g., providers required to login to different systems to manage clinical care).
- Aim 3 describes conducting 3 one-on-one in-depth interview sessions with different groups of 8-10 parents per session per year and a 4th session in year 5 if saturation is not reached. Unclear whether these are one-on-one key informant interviews vs. focus group sessions; data collected from interviews vs. focus groups will result in different types of findings (in depth discussions about personal experiences in former vs. more generalizations of the group in the latter). Remuneration of $100 for participation in one session is also imbalanced with other remuneration structure for RCT; unless plan is for each participant to complete 3 separate interviews (or up to 4) over time.
- (Minor) Cost effectiveness analyses described in IRB and potential savings discussed as part of dissemination plan, but not part of main study aims.

5. Environment:

Strengths

- Appropriate resources, and facilities described.
- Appropriate data coordination and management as well as capability to conduct trial at proposed sites.

Weaknesses

- None noted by reviewer.

Study Timeline:

Strengths

- Appropriate timeline described.

Weaknesses

- None noted by reviewer.

Protections for Human Subjects:

Unacceptable Risks and/or Inadequate Protections

- Needed description for outlined steps of on-call clinician backup to care coordinator.

Data and Safety Monitoring Plan (Applicable for Clinical Trials Only):

Acceptable

Inclusion Plans:

- Sex/Gender: Distribution justified scientifically
- Race/Ethnicity: Distribution justified scientifically
- For NIH-Defined Phase III trials, Plans for valid design and analysis: Not applicable
- Inclusion/Exclusion Based on Age: Distribution justified scientifically

Vertebrate Animals:

Not Applicable (No Vertebrate Animals)

Biohazards:

Not Applicable (No Biohazards)

Resubmission:

- This is an application that was responsive to the prior review. In particular, this revision addressed: translating the app to Spanish, questions about the sensor, once daily vital signs, clarifying power calculations, suggestion to better engage care coordinators and social workers as part of the study protocol, aspects of the design and evaluation, including addressing site-specific differences in racial/ethnic diversity of recruited participants, and tablet provision for those without technology access/WiFi.

Resource Sharing Plans:

Acceptable

Authentication of Key Biological and/or Chemical Resources:

Not Applicable (No Relevant Resources)

Budget and Period of Support:

Budget Modifications Recommended (in amount/time)

Recommended budget modifications or possible overlap identified:

- Given scope of work, concern whether sufficient percent effort budgeted for care coordinator, in particular, and project coordinator at each site.

CRITIQUE 2

Significance: 3
Investigator(s): 2
Innovation: 2
Approach: 6
Environment: 1

Overall Impact: The proposed study would use a randomized controlled trial (RCT) approach to examine the efficacy (6-month period) and sustainability (6-month period) of a previously piloted (for feasibility) mHealth app to support home monitoring for children with complex medical conditions. This app would be used by caregivers. This study would evaluate the impact on outcomes such as child ED/hospital use and quality of life and parental satisfaction, self-efficacy, and stress. The analysis would examine variation in ED/hospital use by race/ethnicity and social determinants of health (SDoH) among app users, and examine factors that shape adherence and sustainability of use of the app. The proposed project has high innovation. Strengths include the population of focus, investigative team, use of mHealth for patients with a range of complex medical conditions, and attention to child healthcare utilization and caregiver outcomes. A moderate weakness in the approach is the lack of a critical analysis of the connection between the mHealth intervention and racial and other disparities, which dampened enthusiasm for this proposed intervention.

1. Significance:

Strengths

- Focus on mHealth technology for children with high health care needs is significant and has promise for impact for this population.

Weaknesses

- The proposal notes that apps can reduce disparities, though it is unclear how the design of the app is designed to reduce disparities.

2. Investigator(s):

Strengths

- Investigative team includes researchers with expertise in implementation science, health information technology, mHealth apps, children with complex medical conditions, qualitative research, and statistics.
- The research team has substantial experience working together, upon which this project would build.

Weaknesses

- The advisory committee appears to be missing some important stakeholders, such as caregivers of children with complex medical conditions.

3. Innovation:

Strengths

- Focus on an mHealth app for a patient population that has variable, multisystem conditions for which remote monitoring holds promise for improving ED/hospitalization use and existing remote monitoring apps are insufficient.
- The focus of remote monitoring on cross-cutting symptoms across this population (for whom conditions may vary) is innovative.
- Focus on understanding the impact of the mHealth app on social factors linked with caregiver and child health, such as caregiver stress is innovative and reflects a holistic approach.
- The assessment of sustainability of use of the mHealth app to monitor subtle health changes that may be precursors to ED/hospitalizations is innovative.

Weaknesses

- None noted by reviewer.

4. Approach:

Strengths

- The study design is well-poised to assess efficacy and sustainability.
- Recruitment approach is appropriate for this population.
- mHealth intervention has been demonstrated to be feasible and daily reporting makes sense both theoretically/conceptually and practically.
- Analytic procedures for quantitative aims are appropriate.

Weaknesses

- Aim 2: It is unclear how the intervention is designed to eliminate inequities affecting racial/ethnic minority/disadvantaged subgroups, particularly since a robust literature indicates that unless technology is specifically designed to address inequities, technological advancements are often poised to exacerbate inequities.
- Aim 2: The examination of disparities related to SDoH assumes that individual factors may drive these persistent disparities, with no articulation of the potential role of structural factors in shaping these outcomes (e.g., bias in health care system).
- Aim 3: The qualitative approach is unclear (e.g., what is the purpose of 2 coders? Will inter-coder agreement be assessed? Will software be used?).

5. Environment:

Strengths

- Research environment is appropriate.

Weaknesses

- None noted by reviewer.

Study Timeline:

Strengths

- None noted by reviewer.

Weaknesses

- Timeline not provided in Research Approach.

Protections for Human Subjects:
Acceptable Risks and/or Adequate Protections

- Adequate protections.

Data and Safety Monitoring Plan (Applicable for Clinical Trials Only):

Acceptable

- Acceptable DSMP.

Inclusion Plans:

- Sex/Gender: Distribution justified scientifically
- Race/Ethnicity: Distribution justified scientifically
- For NIH-Defined Phase III trials, Plans for valid design and analysis: Not applicable
- Inclusion/Exclusion Based on Age: Distribution justified scientifically
- Distributions justified scientifically.

Vertebrate Animals:
Not Applicable (No Vertebrate Animals)

Biohazards:
Not Applicable (No Biohazards)

Resource Sharing Plans:
Not Applicable (No Relevant Resources)

Authentication of Key Biological and/or Chemical Resources:
Not Applicable (No Relevant Resources)

Budget and Period of Support:
Recommend as Requested

CRITIQUE 3

Significance: 1
Investigator(s): 2
Innovation: 1
Approach: 2
Environment: 1

Overall Impact: The current study has high significance because it addresses the need for remote monitoring of symptoms among children with medical complexity (CMC), who have higher health care needs. It will assess the efficacy and sustainability of MyChildCMC app. The app was developed by the PI and pilot tested with high levels of feasibility and acceptability. A 2-arm randomized controlled trial, RCT (MyChildCMC vs. usual care) of CMC (age 1-18 years) and their parents is proposed. To examine reductions in ED/hospital admissions for CMC, by enabling parents, in collaboration with a care coordinator, to better manage their child's condition at home, identifying/responding to early signs of deterioration and seeking/providing timely care when needed. The project is innovative, and the approach is strong. The investigators are experienced and have conducted preliminary studies that adequately support the proposed work. Environment is excellent. Approach has several strengths and only minor weaknesses. Overall, the project remains highly significant.

1. Significance:

Strengths

- Findings from this project will provide an effective, practical, and generalizable approach to supporting home monitoring of children with medical complexity (CMC). This will also serve as a model for reducing recurring emergency department (ED) visits and hospital admissions associated with CMC, generalizable to all children with other multiple chronic conditions.

Weaknesses

- None noted by reviewer.

2. Investigator(s):

Strengths

- The PI of this proposal, has extensive research experience and has a history of successful funding and preliminary work that informs the current proposal, including the creation of the MyChildCMC app
- The rest of the investigative team has complimentary expertise.

Weaknesses

- None noted by reviewer.

3. Innovation:

Strengths

- The focus on CMC is innovative, since as a group these children are often excluded from clinical studies.
- It introduces a novel and generalizable approach to CMC care by focusing on common symptoms (rather than individual underlying conditions) that often precede ED/hospital admissions, making remote CMC monitoring possible despite their multiple and variable underlying conditions.
- It uses an FDA approved wearable sensor, to integrate with the app through Bluetooth and allows automated and accurate recording of objective vital signs data and minimizes data collection burden.

Weaknesses

- None noted by reviewer.

4. Approach:

Strengths

- 3-month pilot trial of 50 CMC randomized to MyChildCMC (n=24) or usual care (n=26) showed feasibility (high participation, adherence and retention), ability to detect early signs of crosscutting symptoms 2-14 days prior to most ED/hospital admissions, fewer hospital days, and higher parent satisfaction.
- The proposed RCT tests the efficacy in a 6-month trial of parents randomly assigned to MyChildCMC or usual care. At the 6-month mark, incentives stopped to assess sustainability over the next 6-month period.
- Evaluation of social determinants of health (SDOH) and outcomes by race/ethnic group to assess additional barriers faced my minoritized populations.
- Assessment of effect of the app on parental/caregiver anxiety.

Weaknesses

- There is the potential for difficulties in the use of the app, especially by parents with low levels of digital literacy. However, pilot data demonstrates high levels of usability for the app and a 100% use of smartphones by parents in the pilot. (minor)

5. Environment:

Strengths

- Excellent environment with an established CMC program: the comprehensive Care Program located at Primary Children's Hospital (PCH) and Riverton Hospital in the Salt Lake City region.
- Research participant recruitment will occur through the LCH's Health Partners Care Coordination (LCHPCC) and Complex Care Program which spans the main hospital site, primary care clinics, and pediatric subspecialty clinics in diverse locations across the downtown, city, and surrounding metropolitan area.

Weaknesses

- None noted by reviewer.

Study Timeline:

Strengths

- Adequate timeline for the proposed research activities.

Weaknesses

- None noted by reviewer.

Protections for Human Subjects:

Acceptable Risks and/or Adequate Protections

Data and Safety Monitoring Plan (Applicable for Clinical Trials Only):

Acceptable

Inclusion Plans:

- Sex/Gender: Distribution justified scientifically
- Race/Ethnicity: Distribution justified scientifically
- For NIH-Defined Phase III trials, Plans for valid design and analysis:
- Inclusion/Exclusion Based on Age: Distribution justified scientifically

Vertebrate Animals:

Not Applicable (No Vertebrate Animals)

Biohazards:

Not Applicable (No Biohazards)

Resubmission:

- The investigators adequately addressed prior reviewers' comments by including Spanish study materials to increase diversity in their sample and equity in research participation for children of Spanish-speaking families and low socioeconomic background, providing explanations on the utility of monitors in younger age groups and in sample size calculations.

Resource Sharing Plans:

Not Applicable (No Relevant Resources)

Authentication of Key Biological and/or Chemical Resources:

Not Applicable (No Relevant Resources)

Budget and Period of Support:

Recommend as Requested

THE FOLLOWING SECTIONS WERE PREPARED BY THE SCIENTIFIC REVIEW OFFICER TO SUMMARIZE THE OUTCOME OF DISCUSSIONS OF THE REVIEW COMMITTEE, OR REVIEWERS' WRITTEN CRITIQUES, ON THE FOLLOWING ISSUES:

PROTECTION OF HUMAN SUBJECTS: UNACCEPTABLE

- Reviewer #1 expressed concern that specific steps in arranging for an on-call clinician back-up for care coordinators at each site are not outlined.
- The panel noted that the Protection of Human Subjects component of the application does align with changes made in the resubmission.

INCLUSION OF WOMEN PLAN: ACCEPTABLE

INCLUSION OF MINORITIES PLAN: ACCEPTABLE

INCLUSION ACROSS THE LIFESPAN: ACCEPTABLE

SCIENTIFIC REVIEW OFFICER'S NOTES: Only information requested in the Notice of Funding Opportunity (NOFO) should be provided in Section 5 of the 'PHS Human Subjects and Clinical Trials Information.' The materials included in Section 5 of this application appear to be Appendix material and should be compliant with the NIH Appendix Policy (NOT-OD-18-126).

COMMITTEE BUDGET RECOMMENDATIONS: The panel expressed concern that the budget did not include adequate effort for the care coordinators and site coordinators at each site.

Footnotes for 1R01NR020784-01A1; PI Name: Nkoy, Flory L

Ad hoc or special section application percentiled against "Total CSR" base.

NIH has modified its policy regarding the receipt of resubmissions (amended applications).See Guide Notice NOT-OD-18-197 at https://grants.nih.gov/grants/guide/notice-files/NOT-OD-18-197.html. The impact/priority score is calculated after discussion of an application by averaging the overall scores (1-9) given by all voting reviewers on the committee and multiplying by 10. The criterion scores are submitted prior to the meeting by the individual reviewers assigned to an application, and are not discussed specifically at the review meeting or calculated into the overall impact score. Some applications also receive a percentile ranking. For details on the review process, see http://grants.nih.gov/grants/peer_review_process.htm#scoring.

MEETING ROSTER

Center for Scientific Review Special Emphasis Panel
CENTER FOR SCIENTIFIC REVIEW
Topics in Health Services Research

ZRG1 HSS-B (90)
07/11/2023 - 07/12/2023

Notice of NIH Policy to All Applicants: Meeting rosters are provided for information purposes only. Applicant investigators and institutional officials must not communicate directly with study section members about an application before or after the review. Failure to observe this policy will create a serious breach of integrity in the peer review process, and may lead to actions outlined in NOT-OD-22-044 at https://grants.nih.gov/grants/guide/notice-files/NOT-OD-22-044.html, including removal of the application from immediate review.

CHAIRPERSON(S)

LYNCH, FRANCES L, PHD, MSPH
SENIOR INVESTIGATOR
CENTER FOR HEALTH RESEARCH
KAISER FOUNDATION RESEARCH INSTITUTE
PORTLAND, OR 97227

MEMBERS

ADAMS, ESTHER KATHLEEN, PHD, MS
PROFESSOR
DEPARTMENT OF HEALTH POLICY AND MANAGEMENT
EMORY UNIVERSITY
ROLLINS SCHOOL OF PUBLIC HEALTH
ATLANTA, GA 30322

ARIAS, JALAYNE J, JD, MA
ASSOCIATE PROFESSOR
DEPARTMENT OF HEALTH POLICY AND
BEHAVIORAL SCIENCES
GEORGIA STATE UNIVERSITY
ATLANTA, GA 30302

ARORA, KAVITA SHAH, MD, MBE, MS
ASSOCIATE PROFESSOR
DIVISION OF GENERAL OBSTETRICS, GYNECOLOGY,
AND MIDWIFERY
UNIVERSITY OF NORTH CAROLINA – CHAPEL HILL
CHAPEL HILL, NC 27599

ATHREYA, ARJUN PRASANNA, PHD, MS
ASSISTANT PROFESSOR
DEPARTMENT OF MOLECULAR PHARMACOLOGY AND
EXPERIMENTAL THERAPEUTICS
MAYO CLINIC
ROCHESTER, MN 55905

BARR, PAUL JAMES, PHD, MSC
ASSOCIATE PROFESSOR
DARTMOUTH INSTITUTE FOR HEALTH POLICY AND
CLINICAL PRACTICE
DARTMOUTH COLLEGE
LEBANON, NH 03756

BARTH, KELLY S, DO
PROFESSOR
DEPARTMENT OF PSYCHIATRY AND
INTERNAL MEDICINE
MEDICAL UNIVERSITY OF SOUTH CAROLINA
CHARLESTON, SC 29425

BOXER, PAUL, PHD
PROFESSOR
DEPARTMENT OF PSYCHOLOGY
RUTGERS UNIVERSITY
NEWARK, NJ 07102

CHAVEZ, LIGIA M, PHD
ASSOCIATE PROFESSOR
BEHAVIORAL SCIENCES RESEARCH INSTITUTE
UNIVERSITY OF PUERTO RICO
MEDICAL SCIENCES CAMPUS
SAN JUAN, PR 00936

DREW, JULIA AR, PHD, MA
RESEARCH SCIENTIST
INSTITUTE FOR SOCIAL RESEARCH & DATA INNOVATION
UNIVERSITY OF MINNESOTA
MINNEAPOLIS, MN 55455

JIMENEZ, NATHALIA, MD, MPH
PROFESSOR
DEPARTMENT OF ANESTHESIOLOGY AND PAIN MEDICINE
UNIVERSITY OF WASHINGTON
SEATTLE, WA 98115

JOHNSON, KAREN A, PHD, MSW
ASSISTANT PROFESSOR
THE SCHOOL OF SOCIAL WORK
UNIVERSITY OF ALABAMA
TUSCALOOSA, AL 35487

JUNG, HYE-YOUNG, PHD
ASSOCIATE PROFESSOR
DEPARTMENT OF POPULATION HEALTH SCIENCES
WEILL CORNELL MEDICAL COLLEGE
NEW YORK, NY 10065

KAPLAN, CAMERON MAXWELL, PHD, MA
ASSOCIATE PROFESSOR
GEHR CENTER FOR HEALTH SYSTEMS
SCIENCES AND INNOVATION
UNIVERSITY OF SOUTHERN CALIFORNIA
LOS ANGELES, CA 90033

KAPOOR, ALOK, MD, MSC
ASSOCIATE PROFESSOR
DEPARTMENT OF MEDICINE
UNIVERSITY OF MASSACHUSETTS
WORCESTER, MA 02118

KIM, HYUNJEE, PHD, MS
ASSOCIATE PROFESSOR
DEPARTMENT OF EMERGENCY MEDICINE
OREGON HEALTH AND SCIENCE UNIVERSITY
PORTLAND, OR 97239

LAYTON, TIMOTHY, PHD
ASSOCIATE PROFESSOR
DEPARTMENT OF HEALTH CARE POLICY
HARVARD UNIVERSITY MEDICAL SCHOOL
BOSTON, MA 02115

LEBRON, ALANA M.W, PHD, MS
ASSISTANT PROFESSOR
DEPARTMENTS OF HEALTH, SOCIETY & BEHAVIOR AND
CHICANO/LATINO STUDIES
UNIVERSITY OF CALIFORNIA IRVINE
IRVINE, CA 92697

MARCELL, ARIK V, MD, MPH
ASSOCIATE PROFESSOR
DEPARTMENT OF PEDIATRICS, POPULATION,
FAMILY AND REPRODUCTIVE HEALTH
JOHNS HOPKINS UNIVERSITY
BALTIMORE, MD 21287

MCCULLOH, RUSSELL JAMES, MD
PROFESSOR
DEPARTMENT OF PEDIATRICS
UNIVERSITY OF NEBRASKA MEDICAL CENTER
OMAHA, NE 681144

MCHUGH, MATTHEW D, PHD, JD, MPH, RN
INDEPENDENCE CHAIR FOR NURSING EDUCATION AND
PROFESSOR
SCHOOL OF NURSING
UNIVERSITY OF PENNSYLVANIA
PHILADELPHIA, PA 19104

MILLER, EMILY STINNETT, MD, MPH
ASSOCIATE PROFESSOR
DIVISION OF MATERNAL FETAL MEDICINE
DEPARTMENT OF OBSTETRICS AND GYNECOLOGY
BROWN UNIVERSITY
PROVIDENCE, RI 02903

MOLFENTER, TODD DAVID, PHD
SENIOR SCIENTIST
CENTER FOR HEALTH ENHANCEMENT SYSTEMS STUDIES
COLLEGE OF ENGINEERING
UNIVERSITY OF WISCONSIN - MADISON
MADISON, WI 53706

NEWCOMER, SOPHIA RAFF, PHD, MPH
ASSOCIATE PROFESSOR
SCHOOL OF PUBLIC AND COMMUNITY HEALTH SCIENCES
UNIVERSITY OF MONTANA
MISSOULA, MT 59812

OSER, CARRIE BETH, PHD
PROFESSOR
DEPARTMENT OF SOCIOLOGY
UNIVERSITY OF KENTUCKY
LEXINGTON, KY 40506

PALATNIK, ANNA, MD
ASSOCIATE PROFESSOR AND ENDOWED CHAIR
MATERNAL FETAL MEDICINE DIVISION
DEPARTMENT OF OBSTETRICS AND GYNECOLOGY
MEDICAL COLLEGE OF WISCONSIN
MILWAUKEE, WI 53226

PATEL, SUNITA K, PHD, MA
ASSOCIATE PROFESSOR
DEPARTMENT OF POPULATION SCIENCES
CITY OF HOPE COMPREHENSIVE CANCER CENTER
DUARTE, CA 91010

PEET, EVAN DAVID, PHD, MA
ECONOMIST
PARDEE RAND GRADUATE SCHOOL
RAND CORPORATION
PITTSBURGH, PA 15213

PERALTA-CARCELEN, ADA MYRIAM, MD, MPH
PROFESSOR
DEPARTMENT OF PEDIATRICS
SCHOOL OF MEDICINE
UNIVERSITY OF ALABAMA AT BIRMINGHAM
BIRMINGHAM, AL 35233

POGORZELSKA-MAZIARZ, MONIKA, PHD, MPH
ASSOCIATE PROFESSOR
COLLEGE OF NURSING
THOMAS JEFFERSON UNIVERSITY
PHILADELPHIA, PA 19107

PROSPERI, MATTIA, PHD
PROFESSOR AND ASSOCIATE DEAN FOR ARTIFICIAL INTELLIGENCE AND INNOVATION
DEPARTMENT OF EPIDEMIOLOGY
COLLEGE OF PUBLIC HEALTH AND HEALTH PROFESSIONS
UNIVERSITY OF FLORIDA
GAINESVILLE, FL 32610

REYNOLDS, WILLIAM STUART, MD, MPH
ASSOCIATE PROFESSOR
DEPARTMENT OF UROLOGY
VANDERBILT UNIVERSITY MEDICAL CENTER
NASHVILLE, TN 37232

SAMAD, MANAR, PHD, MS
ASSISTANT PROFESSOR
DEPARTMENT OF COMPUTER SCIENCE
TENNESSEE STATE UNIVERSITY
NASHVILLE, TN 37209

STULBERG, DEBRA, MD, MA
PROFESSOR AND CHAIR
DEPARTMENT OF FAMILY MEDICINE
UNIVERSITY OF CHICAGO
CHICAGO, IL 60637

WAGNER, ABRAM LUTHER, PHD, MPH
RESEARCH ASSISTANT PROFESSOR
DEPARTMENT OF EPIDEMIOLOGY
UNIVERSITY OF MICHIGAN
ANN ARBOR, MI 48109

WASHINGTON, TYREASA J, PHD, LCSW
CHILD TRENDS SENIOR PROGRAM AREA DIRECTOR
PROFESSOR
DEPARTMENT OF SOCIAL WORK
UNIVERSITY OF NORTH CAROLINA AT GREENSBORO
GREENSBORO, NC 27412

MAIL REVIEWER(S)

BOCKTING, WALTER O, LP, PHD
PROFESSOR OF MEDICAL PSYCHOLOGY
COLUMBIA UNIVERSITY
NEW YORK, NY 10032

HOWELL, DAVID R, PHD, ATC
ASSISTANT PROFESSOR
DEPARTMENT OF ORTHOPEDICS
UNIVERSITY OF COLORADO
AURORA, CO 80045

KATON, JODIE, PHD, MS
RESEARCH HEALTH SCIENCE SPECALIST
VA GREATER LOS ANGELES HEALTH CARE SYSTEM
LOS ANGELES, CA 900073

SCIENTIFIC REVIEW OFFICER

BAKER, MARY KATE, DRPH
SCIENTIFIC REVIEW OFFICER
CENTER FOR SCIENTIFIC REVIEW
NATIONAL INSTITUTES OF HEALTH
6701 ROCKLEDGE DRIVE
BETHESDA, MD 20892

EXTRAMURAL SUPPORT ASSISTANT

NJOKU, PHILIP C
EXTRAMURAL SUPPORT ASSISTANT
CENTER FOR SCIENTIFIC REVIEW
NATIONAL INSTITUTES OF HEALTH
BETHESDA, MD 20892

Consultants are required to absent themselves from the room during the review of any application if their presence would constitute or appear to constitute a conflict of interest.
