# Supplementary material for: The Efficacy of a Home Monitoring App for Children With Medical Complexity: Protocol for a Randomized Controlled Trial
Source: JMIR Res Protoc. 2026 Oct 22;15:e108485. doi: 10.2196/108485 (PMC13717321; doi:10.2196/108485)
Supplement: Supplementary file 2 [file resprot-v15-e108485-s001.docx]

**APPENDIX C: SURVEY INSTRUMENTS**

**PARENT (PRIMARY CAREGIVER) SOCIO-DEMOGRAPHIC QUESTIONNAIRE**

The demographic questionnaire will assess the following parent information, sex, race/ethnicity, marital status, education level, household income, occupation, family size and primary language spoken.

**Questionnaire:** Please check the box that appropriately describes you:

**Respondent Information**

What is your sex? What is your relationship to the child enrolled in our study? _______________

- Male	Number of years providing care to the child? ________________
- Female

**Race/ethnicity**

What is your race?

- White
- Black or African-American
- Hawaiian or Other Pacific Islander
- Asian or Asian American
- Multiple Races

What is your ethnicity?

- Hispanic
- Non-Hispanic

**Marital status**

- Married
- Divorced
- Widowed
- Separated
- Never been married
- A member of an unmarried couple

**Education completed**

Highest grade or year of school you completed?

- Never attended school or only attended kindergarten
- Grades 1 through 8 (Elementary)
- Grades 9 through 11 (Some high school)
- Grade 12 or GED (High school graduate)
- College 1 year to 3 years (Some college of technical school)
- College 4 years (College graduate)
- Master Degree
- Doctoral Degree
- Professional Degree (MD, JD, etc.)

What is the highest grade or year of school your spouse/partner has completed?

- Never attended school or only attended kindergarten
- Grades 1 through 8 (Elementary)
- Grades 9 through 11 (Some high school)
- Grade 12 or GED (High school graduate)
- College 1 year to 3 years (Some college of technical school)
- College 4 years (College graduate)
- Master Degree
- Doctoral Degree
- Professional Degree (MD, JD, etc.)
- Not applicable

**Household Income**

- Under $25,000
- $25,000 - $39,999
- $40,000 - $49,999
- $50,000 - $74,999
- $75,000 - $99,999
- $100,000 - $124,999
- $125,000 - $149,999
- Over $150,000

**Family**

How many children live in your household who are…

Less than 5 years old?	____

5 through 12 years old? ____

13 through 17 years old? ____

**Language**

What is the primary language spoken in the home?

- English	□ Spanish	□ Other Describe__

**DISABILIY SEVERITY: USING THE FUNCTIONAL STATUS SCALE.**

**ZARIT BURDEN/STRESS INTERVIEW SHORT FORM-12 ITEMS (ZBI-12)**

**Please indicate how often you experience the feelings listed by selecting the number that corresponds to the frequency of these feelings.**

1. Do you feel that because of the time you spend with your child that you don’t have enough time for yourself?
   0. Never
   1. Rarely
   2. Sometimes
   3. Quite frequently
   4. Nearly Always
2. Do you feel stressed between caring for your child and trying to meet other responsibility (work, family)?
   0. Never
   1. Rarely
   2. Sometimes
   3. Quite frequently
   4. Nearly Always
3. Do you feel angry when you are around your child?
   0. Never
   1. Rarely
   2. Sometimes
   3. Quite frequently
   4. Nearly Always
4. Do you feel that your relative currently affects your relationship with family member or friends in a negative way?
   0. Never
   1. Rarely
   2. Sometimes
   3. Quite frequently
   4. Nearly Always
5. Do you feel strained when you are around your child?
   0. Never
   1. Rarely
   2. Sometimes
   3. Quite frequently
   4. Nearly Always
6. Do you feel that your health has suffered because of your involvement with your child?
   0. Never
   1. Rarely
   2. Sometimes
   3. Quite frequently
   4. Nearly Always
7. Do you feel that you don’t have as much of privacy as you would like because of your child?
   0. Never
   1. Rarely
   2. Sometimes
   3. Quite frequently
   4. Nearly Always
8. Do you feel that your social like has suffered because you are caring for your relative?
   0. Never
   1. Rarely
   2. Sometimes
   3. Quite frequently
   4. Nearly Always
9. Do you feel that you have lost control of your life since caring for your child?
   0. Never
   1. Rarely
   2. Sometimes
   3. Quite frequently
   4. Nearly Always
10. Do you feel uncertain about what to do about your child?
    0. Never
    1. Rarely
    2. Sometimes
    3. Quite frequently
    4. Nearly Always
11. Do you feel you should be doing more for your child?
    0. Never
    1. Rarely
    2. Sometimes
    3. Quite frequently
    4. Nearly Always
12. Do you feel you could do a better job in caring for your child?
    0. Never
    1. Rarely
    2. Sometimes
    3. Quite frequently
    4. Nearly Always

**OSLO SOCIAL SUPPORT SCALE (OSSS-3)**

1. How many people are so close to you that you can count on them if you have great personal problems?
   1. None
   2. 1–2
   3. 3–5
   4. 5+

2. Oslo 2: How much interest and concern do people show in what you do?
   1. None
   2. Little
   3. Uncertain
   4. Some
   5. A lot

3. Oslo 3: How easy is it to get practical help from neighbors if you should need it?
   1. Very Difficult
   2. Difficult
   3. Possible
   4. Easy
   5. Very Easy

The sum score ranges from 3 to 14, with high values representing strong levels and low values representing poor levels of social support. This continuous score was used to generate the normative data for the OSSS-3 for each scoring point as well as to determine group differences according to age and sex.

**BRIEF Health Literacy Screening Tool (BRIEF)**

**Please circle the answer that best represents your response.**

1. How often do you have someone help you read clinic or hospital materials?
1. Always
2. Often
3. Sometimes
4. Occasionally
5. Never
2. How often do you have problems learning about your child’s medical condition because of difficulty understanding written information?
   1. Always
   2. Often
   3. Sometimes
   4. Occasionally
   5. Never
3. How often do you have a problem understanding what is told to you about your child’s medical condition?
1. Always
2. Often
3. Sometimes
4. Occasionally
5. Never
4. How confident are you filling out your child’s medical forms by yourself?
   1. Not at all
   2. A little bit
   3. Somewhat
   4. Quite a bit
   5. Extremely

| BRIEF | Score | Skills and Abilities |
| --- | --- | --- |
| Limited | **4-12** | Not able to read most low literacy health materials; will need repeated oral instructions; materials should be composed of illustrations or video tapes. Will need low literacy materials; may not be able to read a prescription label. |
| Marginal | **13-16** | May need assistance; may struggle with patient education materials. |
| Adequate | **17-20** | Will be able to read and comprehend most patient education materials. |

**CHILD’S QUALITY OF LIFE QUESTIONNAIRE (ADAPTED FROM** **ELLZEY et. al. QUESTIONNAIRE)**

**PARENT SATISFACTION QUESTION: ADAPTED FROM CLIENT SATISFACTION QUESTIONNAIRE**

1. I feel very confident in my ability to effectively manage or take good care of my child’s health.
   a. Strongly disagree,
   b. Disagree,
   c. Neither Agree nor Disagree
   d. Agree,
   e. Strongly agree,
   f. Don’t know
2. I am very consistent in doing all the things I need to do to effectively manage my child’s health.
   a. Strongly disagree,
   b. Disagree,
   c. Neither Agree nor Disagree
   d. Agree,
   e. Strongly agree,
   f. Don’t know
3. We have all the medical professional support we need to help with my child’s health issues.
   a. Strongly disagree,
   b. Disagree,
   c. Neither Agree nor Disagree
   d. Agree,
   e. Strongly agree,
   f. Don’t know
4. We have positive social support (e.g., from family, friends, others) to help as needed with my child’s health issues.
   a. Strongly disagree,
   b. Disagree,
   c. Neither Agree nor Disagree
   d. Agree,
   e. Strongly agree,
   f. Don’t know
5. We have a good system or structure in place (e.g. a good plan, schedule for care tasks, a tool etc.) to help in managing or taking good care of my child’s health.
   a. Strongly disagree,
   b. Disagree,
   c. Neither Agree nor Disagree
   d. Agree,
   e. Strongly agree,
   f. Don’t know
6. I don’t feel very stressed or anxious about my child’s health or about my role in managing or taking care of my child’s health.
   a. Strongly disagree,
   b. Disagree,
   c. Neither Agree nor Disagree
   d. Agree,
   e. Strongly agree,
   f. Don’t know
7. Overall, I am satisfied with the care and services my child received.
   a. Strongly disagree,
   b. Disagree,
   c. Neither Agree nor Disagree
   d. Agree,
   e. Strongly agree,
   f. Don’t know

**PARENT CAREGIVING SELF-EFFICACY**

**Parents’ Self-Efficacy in Caring for CMC Survey and in managing their symptoms and needs**

Please indicate how confident you feel in your ability to do the following in caring for your child.

2. Monitoring your child’s various symptoms for trends and changes.
   0. Not at all
   1. Slightly
   2. Somewhat
   3. Very
   4. Extremely
3. Decreasing your child’s pain when it elevates.
0. Not at all
1. Slightly
2. Somewhat
3. Very
4. Extremely
4. Increasing your child’s oxygen saturation levels when they’re low.
0. Not at all
1. Slightly
2. Somewhat
3. Very
4. Extremely
5. Decreasing your child’s temperature when s/he has a fever.
0. Not at all
1. Slightly
2. Somewhat
3. Very
4. Extremely
6. Decreasing your child’s heart rate when it’s elevated.
0. Not at all
1. Slightly
2. Somewhat
3. Very
4. Extremely
7. Reducing the length or severity of your child’s seizures.
0. Not at all
1. Slightly
2. Somewhat
3. Very
4. Extremely
8. Helping your child with any emotional distress or concerns like anxiety s/he has.
0. Not at all
1. Slightly
2. Somewhat
3. Very
4. Extremely
9. Helping your child generally live the healthiest and fullest life s/he can.
0. Not at all
1. Slightly
2. Somewhat
3. Very
4. Extremely
10. Knowing what to do when there is a rapid worsening of one or more of your child’s symptoms.
0. Not at all
1. Slightly
2. Somewhat
3. Very
4. Extremely
11. Knowing when to take your child to the hospital for emergency treatment of her/his symptoms.
0. Not at all
1. Slightly
2. Somewhat
3. Very
4. Extremely
