# Supplementary material for: The Efficacy of a Home Monitoring App for Children With Medical Complexity: Protocol for a Randomized Controlled Trial
Source: JMIR Res Protoc. 2026 Oct 22;15:e108485. doi: 10.2196/108485 (PMC13717321; doi:10.2196/108485)
Supplement: Supplementary file 3 [file resprot-v15-e108485-s002.docx]

**SPIRIT 2025 checklist of items to address in a randomized trial protocol***

| **Section / Topic** | **No** | **SPIRIT 2025 checklist item description** | **Reported on page no.** |
| --- | --- | --- | --- |
| **Administrative information** | | |  |
| Title and structured summary | 1a | **Title stating the trial design, population, and interventions, with identification as a protocol**  The Efficacy of a Home Monitoring App for Children with Medical Complexity: Protocol for a Randomized Controlled Trial | **1** |
|  | 1b | **Structured summary of trial design and methods, including items from the World Health Organization Trial Registration Data Set.** This is a multicenter randomized controlled trial to assess the efficacy and sustainability of MyChildCMC, the first app designed to monitor crosscutting symptoms and identify early signs of health deterioration in children with medical complexity (CMC) to prompt early interventions and prevent recurrent ED/hospital admissions. Study participation will last 12 months and includes 2 parts. The first part is a 6 month, 2-arm (MyChildCMC vs usual care) trial of CMC (age 1-18 years) and their parents. Parents assigned to MyChildCMC use the app daily for 6 months to monitor symptoms. Those assigned to usual care use a paper diary (or logbook) to monitor symptoms. Both arms receive financial incentive for daily monitoring of symptoms (monitoring incentives) and for responding to surveys. We will assess efficacy at 6 months. Then we will stop the monitoring incentive at 6 months, but participants will continue with daily monitoring for 6 more months, after which we will assess sustainability (with daily use and of the intervention effects) in the app group at 12 months. | **1** |
| Protocol version | 2 | **Version date and identifier** Version 3.0_September 2026 | **1** |
| Roles and responsibilities | 3a | **Names, affiliations, and roles of protocol contributors**   1. Flory Nkoy, MD, MS, MPH; University of Utah; Principal Investigator 2. Bryan Stone, MD, MS; University of Utah; co-Investigator 3. Carolyn Foster, MD, MS; Ann & Robert H. Lurie Children’s Hospital (LCH)of Chicago; co-Investigator (Chicago Site Leader) 4. Angela Zhu, BS; University of Utah; Senior Clinical Research Coordinator (CRC) 5. Sara McCormick, MPA; University of Utah; Project Manager 6. Maya Wheeler, MS; University of Utah; Clinical Research Coordinator 7. Peter Walsh, BS; Ann & Robert H. LCH of Chicago (LCH); CRC 8. Connor Karwowski, BS; Ann & Robert H. LCH of Chicago; CRC 9. Savanah East, NP; University of Utah; Care Coordinator 10. Katherine Sward, PhD; University of Utah; co-Investigator 11. Xiaoming Sheng, PhD; University of Utah; co-Investigator 12. Carolyn Reynolds, NP, MS; Intermountain Health; co-Investigator 13. Justin Poll, PhD; Intermountain Health; Interview Facilitator 14. Nancy Murphy, MD: University of Utah; co-Investigator (Utah Site Leader) | **5,6** |
|  | 3b | **Name and contact information for the trial sponsor**  Ronald Wertz (Grants Management Specialist); Phone: 301-594-2807; Email: wertzr@mail.nih.gov | **1** |
|  | 3c | **Role of trial sponsor and funders in design, conduct, analysis, and reporting of trial; including any authority over these activities**: None | **1** |
|  | 3d | **Composition, roles, and responsibilities of the coordinating site, steering committee, endpoint adjudication committee, data management team, and other individuals or groups overseeing the trial, if applicable:** N/A | **N/A** |
| **Open science** | | |  |
| Trial registration | 4 | **Name of trial registry, identifying number (with URL), and date of registration. If not yet registered, name of intended registry.** Clinical Trial.gov; NCT06883045 (<https://register.clinicaltrials.gov/prs/beta/studies/S000FIDW00000039/recordSummary>); 03/12/2025 | **1** |
| Protocol and statistical analysis plan | 5 | **Where the trial protocol and statistical analysis plan can be accessed:** University of Utah UBox | **N/A** |
| Data sharing | 6 | **Where and how the individual de-identified participant data (including data dictionary), statistical code, and any other materials will be accessible.**  Any data shared will be de-identified. All data and resources will be shared following the NIH, University of Utah IRB, Lurie Children’s Hospital’s, and Intermountain Healthcare’s policies. A Data Sharing Agreement will be required that will describe the conditions and restrictions of their use; limited data access will be made available only to users who successfully complete a rigorous approval process by both the investigators and the community partners. Specifically, we will make the data and associated documentation available to users only under data-sharing agreement that include: 1) a commitment to using the data only for research purposes and not to identify any individual participant; 2) a commitment to securing the data using an appropriate method; and 3) a commitment to destroying the data after analyses are completed. Data sets will be encrypted for transfer to approved investigators.  The dataset will include an associated metadata file with detailed descriptions of all of the variables, investigator contact information, dates of data collection, geographic location of data collection, and information about funding sources. Other resources developed will also be shared including study protocol, survey instruments and key informant interview guides developed by the research team. After the results from this study are published, we will make survey instruments, key informant interview, and focus group guides available to the scientific community with the agreement that they will appropriately acknowledge the research team who developed the instruments. The study team can be readily contacted by email (email addresses are included in the grant application and will be available in any published manuscripts). We anticipate honoring all requests, if requesting individuals identify themselves and the purpose for which the instruments are requested.  We will share deidentified data stored within the database with the scientific community, and no specialized tools, software or code will be needed to access or manipulate shared data. All the data and metadata will be stored within the University of Utah protected environment, accessible only to the University employees who are also part of the investigation team. We anticipate that this will be a small dataset (up to 2 GB in size) and will be included as supplementary material to accompany articles submitted to PubMed Central. Data will be archived in The Hive for sharing after study publication. The Hive is a publicly-accessible repository for research data generated by University of Utah researchers, students, and staff. It is maintained by the Marriott Library IT and Digital Collections. The Hive has policies/procedures in place that provide data access to qualified researchers, fully consistent with NIH data sharing policies and applicable laws and regulations.  Researchers can directly contact the research team by email to request and the research team will send them the requested data. The data will be identifiable via a persistent unique identifier. Every dataset submitted to The Hive receives a Digital Object Identifier (DOI) and suggested data citation to submit with publication manuscripts. The DOI facilitates linking between publications and the underlying datasets. We will make the data available to the research community when the award ends. Three years after the completion of the study and publication of results, all data will be destroyed.  We agree with the NIH data-sharing policy, in that data from human subjects derived from our study will be shared only if the identity and privacy of the research participants can be protected. We will work closely with the NIH to ensure full compliance with all requirements while preserving the privacy of individual participants. Data sets released will be stripped of all PHI to allow sharing of data without compromising participant confidentiality, privacy, and safety. Data will be stripped of identifiers that would permit linkages to individual research participants and will exclude variables that could lead to deductive disclosure of the identity of individual subjects. De-identification of data will be meticulously completed with awareness that our participants’ privacy must be protected in agreement with all applicable laws and regulations. Research staff will create de-identified datasets and accompanying documentation (data dictionaries, annotated forms, and manuals) to be used for data sharing. Thus, the rights and privacy of research participants will be protected at all times during data sharing. We will work with the study statistician, to accomplish this. All identification covered under HIPAA will be removed. All remaining variables not covered under HIPAA will be evaluated in terms of the risk of deductive disclosure of identity, and appropriate measures will be taken to protect confidentiality. The striped data, in partnership with the study partners, will be sent to the NIH Program Officer for public release. In order to ensure responsible usage of study data, user registration will be required to access or download data files. | **22-24** |
| Funding and conflicts of interest | 7a | **Sources of funding and other support (e.g., supply of drugs):** NIH | **1** |
|  | 7b | **Financial and other conflicts of interest for principal investigators and steering committee members:** None | **N/A** |
| Dissemination policy | 8 | **Plans to communicate trial results to participants, healthcare professionals, the public, and other relevant groups (e.g., reporting in trial registry, plain language summary, publication).** This study is a clinical trial. We registered the study and will submit results to ClinicalTrials.gov as required by NIH policy. The University of Utah, LCH and Intermountain each have internal policies to ensure that clinical trial registration and results reporting comply with NIH requirements. We will disseminate quantitative and qualitative results, the study protocol, significant findings, the technical approach, implementation methods, analytic methods, and final study data through peer-reviewed publications and presentations at national conferences. | **24** |
| **Introduction** | | |  |
| Background and rationale | 9a | **Scientific background and rationale, including summary of relevant studies (published and unpublished) examining benefits and harms for each intervention.** CMC are the most vulnerable of children with chronic diseases, who have complex, multisystem chronic diseases affecting ≥3 organ systems, severe functional limitations and technology dependencies. CMC have high health care needs, and account for 40% of hospitalized children and 35% of all pediatric health care costs. Due to high medical fragility, CMC have frequent acute deteriorations superimposed on their chronic conditions, leading to recurrent ED/hospital admissions and QOL. To reduce ED/hospital admissions for CMC, remote monitoring is suggested, with use of mHealth apps to regularly assess their health status remotely and identify early signs of acute deterioration, allowing for early interventions to prevent ED/hospital admissions. Yet no app exist to support CMC remote monitoring. Variable, multisystem conditions among CMC make it difficult to develop an app. Also, parental factors can affect CMC’s health outcomes, but the impacts of these factors on the intervention effects of a monitoring app for CMC are rarely studied. Fortunately, ED/hospital admissions for CMC are often preceded by a limited set of shared (crosscutting) acute symptoms. These crosscutting symptoms rarely occur suddenly. Studies suggest that they usually start as subtle signs, often unnoticed by parents until they escalate to prompt an ED/hospital visit. Thus, crosscutting symptoms offer an opportunity for a novel and practical approach for developing a remote monitoring app for CMC, despite their multiple, variable underlying conditions. In a focus group, parents identified the crosscutting symptoms that most often preceded their children’s hospital admissions, and conveyed their needs, preferences and key functionalities that led to MyChildCMC, the first app designed to monitor and identify early signs of crosscutting symptoms in CMC. In a pilot trial of 50 subjects, we confirmed feasibility of MyChildCMC use by parents, ability to detect early signs 2-14 days prior to ED/hospital admissions, and use leading significantly to fewer hospital days than controls. The current study will assess the efficacy and sustainability of MyChildCMC in a fully-powered 6-month, 2-arm trial of CMC (age 1-18 years) and their parents. Parents assigned to MyChildCMC will use the app daily for 6 months, both arms will receive financial incentive for participation, then we will stop the incentive and follow subjects for 6 more months to assess sustainability at 12 months. We will also assess MyChildCMC’s impact on ED/hospital admissions among different parent subgroups. | **8-9** |
|  | 9b | **Explanation for choice of comparator.** The comparator (Usual Care) is the care CMC typically receive. | **9** |
| Objectives | 10 | **Specific objectives related to benefits and harms:**  **1)** Determine MyChildCMC’s efficacy on **1.a.** Child (ED/hospital use, hospital days and QOL) and **1.b.** Parent (satisfaction, self-efficacy and stress) outcomes.  **2)** Determine sustainability of MyChildCMC use and of the intervention effects. | **9** |
| **Methods: Patient and public involvement, trial design** | | |  |
| Patient and public involvement | 11 | **Details of, or plans for, patient or public involvement in the design, conduct, and reporting of the trial** | **N/A** |
| Trial design | 12 | **Description of trial design including type of trial (e.g., parallel group, crossover), allocation ratio, and framework (e.g., superiority, equivalence, non-inferiority, exploratory)**. A 6-month, 2-arm (parallel) trial (MyChildCMC vs usual care) of CMC (age 1-18 years) and their parents. Parents assigned to MyChildCMC use the app daily for 6 months. Those assigned to usual care use a paper diary daily for 6 months. Both arms receive financial incentive for daily monitoring (monitoring incentive) for 6-months and for responding to surveys. We will assess efficacy at 6 months. Then we will stop the monitoring incentive at 6 months and follow subjects for 6 more months to assess sustainability of the app at 12 months. | **9** |
| **Methods: Participants, interventions, and outcomes** | | |  |
| Trial setting | 13 | **Settings (e.g., community, hospital) and locations (e.g., countries, sites) where the trial will be conducted.** The study is being conducted at 2 sites: 1) Salt Lake City (SLC), Utah, and 2) Chicago, IL. SLC site includes the Pediatric Comprehensive Care Program (CCP) for CMC, located in the ambulatory section of Primary Children’s Hospital (PCH), University of Utah affiliated. Chicago site includes LCH outpatient clinic for CMC. LCH is a Northwestern University Feinberg School of Medicine's affiliated pediatric hospital. | **2** |
| Eligibility criteria | 14a | **Eligibility criteria for participants**  Inclusion criteria: 1) CMC receiving care at the 2 sites, 2) CMC’s age 1-18 years, 3) Parent willing to use their smartphone (or tablet) and internet to participate, and 4) Parent speaks English or Spanish. CMC are children having a combination [(a, b, AND c) or (d AND (a or b or c))] of: a) multisystem chronic diseases involving ≥3 organ systems, b) ≥3 health care providers involved in their care, c) high fragility or frequent (≥2) or 1 prolonged (>10 days) hospitalization in the prior year, and/or d) medical technology dependence.  Exclusion criteria: CMC are excluded if they are: 1) >18 years of age, 2) Critically ill, 3) Infant, 4) In hospice without concurrent care, or 5) Not English or Spanish speakers. Infants are excluded as many CMC <1 year have prematurity-related conditions that may be resolved by age 2. CMC >18 years are excluded as some transition to adult care. Note: To reduce the digital divide among CMC parents, we are providing families without smartphone or internet low-cost tablets and cellular internet to allow them to participate. We estimated ~10% of CMC parents will need this support. | **2** |
|  | 14b | **If applicable, eligibility criteria for sites and for individuals who will deliver the interventions (e.g., surgeons, physiotherapists)** | **N/A** |
| Intervention and comparator | 15a | **Intervention and comparator with sufficient details to allow replication including how, when, and by whom they will be administered. If relevant, where additional materials describing the intervention and comparator (e.g., intervention manual) can be accessed**  Intervention (MyChildCMC) is an app designed to support remote monitoring of CMC crosscutting symptoms to identify signs of CMC’s health deterioration early to change their care early to avoid progression and prevent ED/hospital admissions. The app includes the following features: 1) automated reminders, 2) vital sign/symptom monitoring, 3) real-time feedback system, 4) automated and real-time summary reports with longitudinal graphs, 5) automated alerts to a care coordinator, and 6) clinic dashboard connecting patients to clinics and allowing access to patient data to support care coordination.  Comparator (Usual Care) includes the following services: 1) Develop a shared care plan for the child; 2) Parent education on potential interventions to try at home if a child parameter is abnormal (e.g. feeding intolerance, pain, etc.); 3) Social worker’s consultation and connection to community resources; 4) Care coordination of medical care, referrals, and appointments, in collaboration with health care providers (HCPs); 5) Inpatient consultation for hospitalized CMC. Also, many parents track their child’s vital signs/symptoms and medications on paper as part of usual care. | **1, 10-12** |
|  | 15b | **Criteria for discontinuing or modifying allocated intervention/comparator for a trial participant (e.g., drug dose change in response to harms, participant request, or improving/worsening disease)**  N/A. This a low-risk intervention. There are no criteria for discontinuing or modifying allocated intervention/comparator for a trial participant. | **N/A** |
|  | 15c | **Strategies to improve adherence to intervention/comparator protocols, if applicable, and any procedures for monitoring adherence (e.g., drug tablet return, sessions attended)**  Participants will receive monetary incentives to improve adherence to the intervention and protocols. Those randomized to MyChildCMC will receive $130 for daily use of MyChildCMC app ($10 every 2 weeks for 6 months, or about 13 payments) to monitor symptoms. Usual care group receive same incentives ($130 total) for monitoring symptoms on paper and submitting images of completed logbook pages. All participants will receive $50 for completing each survey: baseline, 3, 6, and 12-mo surveys ($200 for surveys). Overall, each participant will receive $330 total if they complete all study activities. | **2, 4, 9, 11, 13** |
|  | 15d | **Concomitant care that is permitted or prohibited during the trial** | **N/A** |
| Outcomes | 16 | **Primary and secondary outcomes, including the specific measurement variable (e.g., systolic blood pressure), analysis metric (e.g., change from baseline, final value, time to event), method of aggregation (e.g., median, proportion), and time point for each outcome**   - Primary outcome: ED/hospital admissions (12 months pre- and 12 months post-enrollment) or number of ED/hospital admissions for the CMC, 12 months pre and 12 months post enrollment. - Secondary outcomes:   - Child’s quality of life (QOL), collected at baseline, 3, 6 and 12 months.   - Hospital days (12 months pre- and 12 months post enrollment)   - Parent stress, collected at baseline, 3, 6 and 12 months   - Caregiving self-efficacy (in managing child’s symptoms), collected at baseline, 3, 6 and 12 months.   - Parent satisfaction with child’s care, collected at baseline and 6 months | **3, 12, 13** |
| Harms | 17 | **How harms are defined and will be assessed (e.g., systematically, non-systematically)**  Unanticipated harms will be recorded in a data collection form at each follow-up survey. We will assess occurrence of adverse events (AEs)/serious adverse events (SAEs) and record all events with likelihood of association with *MyChildCMC* use, start dates and outcomes. Risks described in the protocol and consent form do not have to be reported as SAEs, unless the expected event occurs more frequently or is more serious than expected. One exception to this rule is in the case of a death. All deaths must be reported, if or not it was related to the intervention.  The *MyChildCMC* intervention amounts to an improved communication tool including provision of objective and subjective longitudinal data to be monitored by parents and care coordinators (and physicians). While vital signs (temp, HR, RR and O2Sat) will be collected objectively, it is possible that parents will enter erroneous symptoms data (e.g. feeding intolerance, pain, mental status, etc.), care coordinators and physicians will be cautioned through app pop-up reminders of this fact and advised to avoid relying solely on the *MyChildCMC* data in their medical decision-making. Although we don’t anticipate safety events, our monitoring will also include evaluation for poor outcomes including suggestion of a delay in care [e.g., arrival at the ED in an extreme condition, or admission directly to the pediatric ICU), and potential resource overuse (increased ED/hospital admissions for acute healthcare concerns) driven by changes in vital signs/symptoms not associated with acute changes in the child’s health. Should we note an event suggesting a delay or overuse in care, we will discuss the potential influence of the *MyChildCMC* with the involved clinic staff and report events that may represent safety events immediately to the ISM, IRB and if deemed necessary to the NINR, for action taken by the IRB.  AEs that take place during interviews or focus groups will be collected and monitored by study personnel. SAEs and non-SAEs that occur outside of research-related activities will be monitored using a brief questionnaire administered to parents at all data collection time points using a study-specific form tailored for the project. All AEs will be reported to the PI and research team who will review them in relation to the study protocol to assess the likelihood that the AE is study-related, and the risk for recurrence. The AE form will include the following items: date and description of event; assessment as to whether the event is an AE; if yes, whether it is a SAE; whether it is anticipated or unanticipated; and whether it is related or unrelated to the project. An event will be considered a SAE if it results in death, is life-threatening, requires inpatient hospitalization, results in a persistent or significant disability/ incapacity, or might require medical intervention to prevent one of these outcomes. Non-SAEs are conditions that may be unpleasant but that would not require termination of participation in the program. SAEs will be reported to the University of Utah SIRB within 5 business days. Non-SAEs will be reported to the IRB in a summary report and will specify the nature and frequency of the AEs. Yet any non-SAEs that are deemed an “unanticipated problem” will be reported to the IRB on the same schedule as a SAE. | **19-22** |
| Participant timeline | 18 | **Time schedule of enrollment, interventions (including any run-ins and washouts), assessments, and visits for participants. A schematic diagram is highly recommended (see Figure)**  Study Diagram   | **22** |
| Sample size | 19 | **How sample size was determined, including all assumptions supporting the sample size calculation**  Sample Size Calculation for the Primary Objective: Our pilot data showed a pooled group baseline rate of 0.98 per 3 person-months for the primary outcome (ED/hospital admissions) or a background rate of 1.96 per 6 person-months of our target follow-up time. We will enroll 360 participants (dyads), with an estimated 15%~20% loss to follow-up, leading to 288 ~306 subjects completing the study. Assuming an average pre-post correlation of at least 0.5 on the primary outcome which diminishes the sample size by 1.5 as a design effect, we will have equivalent sample sizes of 384~408 subjects over the 2 time periods. A Poisson regression of ED/hospital admissions with a sample of 384 subjects (20% loss to follow-up) achieves 80% power to detect a response rate ratio (RR) of 0.744 or less for the intervention vs control arms, at 0.05 significance level. If there are other standardized variables being adjusted in the model, where a multiple regression of the control/intervention group on other adjusted covariates is expected to have an R-Squared (R^2^) of 0.20, the detectable RR will be 0.717. With 408 total subjects completing the study (15% loss to follow-up), the detectable RR will be 0.751, and if there are other standardized variables being adjusted in the model with an R^2^ of 0.20, the detectable RR will be 0.725. | **18-19** |
| Recruitment | 20 | **Strategies for achieving adequate participant enrollment to reach target sample size**  We are using 2 ways to enroll participants including face-to-face (at the time of clinic visit or when participants are hospitalized) or remote enrollment approaches. We are also submitting an amendment to add the following: 1) Distributing and/or posting flyers at participating clinics; 2) Adding a QR code to flyers for patients to complete a brief survey expressing interest; 3) Adding a brief survey to be handed out by clinic front staff with their clinic paperwork to ask about study interest; and 4) Adding remote recruitment options: emailing and sending letters in the mail to pre-screened participants. *In addition, we are using multiple strategies to support retention, including giving financial incentives, mailing thank you and birthday cards, and confirming contact information at each follow-up survey. To increase recruitment/retention of minorities, low literacy and underserved participants, we will use a variety of evidence-based practices such as using low-literacy recruitment materials and scripts, face-to-face recruitment if possible, developing rapport and having regular communication with families, providing timely gift card incentive, and using a research staff from the same racial/ethnic background.* | **14-16** |
| **Methods: Assignment of interventions** | | |  |
| Randomization: |  |  |  |
| Sequence generation | 21a | **Who will generate the random allocation sequence and the method used**  Random allocation will be automatically generated using REDCap. | **2, 12** |
|  | 21b | **Type of randomization (simple or restricted) and details of any factors for stratification. To reduce predictability of a random sequence, other details of any planned restriction (e.g., blocking) should be provided in a separate document that is unavailable to those who enroll participants or assign interventions**. A computer-generated allocation sequence is built into REDCap to help with randomization, which is stratified by age group (1-11 vs 12-18) and child disability severity (6-15 vs 16-30) as they may affect child outcomes. Child disability severity is measured at enrollment using the Functional Status Scale, which assesses the child’s functional status across 6 domains with scores ranging from 6 (normal function) to 30 (very severe dysfunction. Participants are allocated into 2 arms in a 1:1 ratio using a block-randomized scheme with random block sizes of either 4 or 6. Once an eligible subject consents and completes baseline surveys, the research team uses REDCap to identify the allocation number (real allocation arm is concealed to the study teams) and then discloses it to the subject. | **2, 12** |
| Allocation concealment  mechanism | 22 | **Mechanism used to implement the random allocation sequence (e.g., central computer/telephone; sequentially numbered, opaque, sealed containers), describing any steps to conceal the sequence until interventions are assigned.** A computer-generated allocation sequence is incorporated into the Study’s REDCap database to facilitate randomization. Participants are allocated into 2 arms in a 1:1 ratio using a block-randomized scheme with random block sizes of either 4 or 6. | **2, 12** |
| Implementation | 23 | **Whether the personnel who will enroll and those who will assign participants to the interventions will have access to the random allocation sequence**. None one has access to the random allocation sequence. Random allocation is automatically generated using REDCap. | **2, 12** |
| Blinding | 24a | **Who will be blinded after assignment to interventions (e.g., participants, care providers, outcome assessors, data analysts). No** blinding is used. | **2, 12** |
|  | 24b | **If blinded, how blinding will be achieved and description of the similarity of interventions** | **N/A** |
|  | 24c | **If blinded, circumstances under which unblinding is permissible, and procedure for revealing a participant’s allocated intervention during the trial** | **N/A** |
| **Methods: Data collection, management, and analysis** | | |  |
| Data collection methods | 25a | **Plans for assessment and collection of trial data, including any related processes to promote data quality (e.g., duplicate measurements, training of assessors) and a description of trial instruments (e.g., questionnaires, laboratory tests) along with their reliability and validity, if known. Reference to where data collection forms can be accessed, if not in the protocol.** We will collect demographics (including parent education level), health literacy and parent social support will be collected for both arms at baseline (enrollment), at clinic or online using REDCap. Parent social support will be collected using Oslo social support scale (OSSS-3), a 3-item self-reported measure, with scores ranging from 3 (lowest) to 14 (highest). Both arms will complete surveys (child’s QOL, parent caregiving self-efficacy and stress) at baseline, 3, 6 and 12 months, and parent satisfaction (baseline and 6 months). For those randomized to MyChildCMC, at the first login, after completing baseline surveys, the RC will guide them to complete the first assessment of crosscutting symptoms. Child’s QOL will be collected using a CMC specific survey validated by Ellzey et. al, (Dr. Murphy, Co-I, was a co-author). The 7-item survey assesses physical health, mental health, sleep, pain, activities and general QOL. Health literacy will be collected using the Brief Health Literacy Screening Tool, a 4-item survey, with scores ranging from 4 to 20 (4-12=limited, 13-16=Marginal, 17-20=Adequate literacy). Caregiver stress will be assessed using the Zarit Burden Interview (ZBI-12), a self-administered 12-item validated survey, with 5 responses ranging from 0 (never) to 4 (always). It assesses stress/coping, negative affect, social support, with a total score rating care-giving stress (burden) as no/little (0-2), mild-to-moderate (3-7), moderate-to-severe (8-18) and high/severe (>18). Self-efficacy will be collected using a CMC specific questionnaire adapted from validated surveys, including the PROMIS and PhenX Toolkit self-efficacy scales, as no existing scale meets CMC needs. Parent satisfaction will be collected using questions adapted from Client Satisfaction Questionnaire. Follow-up surveys will be collected using REDCap at 3, 6 and 12 months. ED/ hospital admissions and hospital days (6 months pre/post-enrollment (for impact analysis) and 7-12 months post-enrollment (for sustainability) will be collected electronically using the Intermountain enterprise data warehouse (EDW), an integrated database that links data from 22 hospitals and 180 clinics in Utah, and the All Payor Claims Database, capturing health care services for ~100% many CMC in Utah, and LCH EDW, which captures 70-80% of CMC health services in Chicago. To supplement the LCH EDW’s data, we will use the Chicago Area Patient-Centered Outcomes Research Network (CAPriCORN) data, which stores data from 11 healthcare systems/ hospitals in Chicago, and will help capture most of the utilization data occurring outside LCH systems. | **3, 4, 12** |
|  | 25b | **Plans to promote participant retention and complete follow-up, including list of any outcome data to be collected for participants who discontinue or deviate from intervention protocols**  We will use multiple strategies to support retention, including giving financial incentives, mailing thank you and birthday cards, and confirming contact information at each follow-up survey. To increase recruitment/retention of minorities, low literacy and underserved participants, we will use a variety of evidence-based practices such as using low-literacy recruitment materials and scripts, face-to-face recruitment if possible, developing rapport and having regular communication with families, providing timely gift card incentive, and using a research staff from the same racial/ethnic background. Potentially eligible participants who decide to opt out of trial participation will have no data collected. Patients who initially enroll in the trial and later elect to withdraw from the trial will have no data collected after the date of withdrawal. If a child is placed on end-of-life care, they will be withdrawn from the study (for both arms). Data collected prior to withdrawal will remain in the trial database. Contact information for the research personnel will be available at participating sites clinics throughout the duration of the trial to facilitate communication such as a decision to withdraw from the trial. | **19** |
| Data management | 26 | **Plans for data entry, coding, security, and storage, including any related processes to promote data quality (e.g., double data entry; range checks for data values). Reference to where details of data management procedures can be accessed, if not in the protocol**  The proposed project will include 360 children with medical complexity (CMC) and their parents (or primary caregivers) who are screened and enrolled in the study to use *MyChildCMC* app or are in the control group (usual care). Sharing data is an essential part of our proposed project activities and will help make our results available to clinicians, scientists and other stakeholders interested in improving quality of life (QOL) and reducing ED and hospital admissions in CMC.  Thus, data generated from our project will include data collected, via the app or diary, directly from CMC and their parents (or primary caregivers). The app includes data quality and validation check to ensure that vital signs and symptoms collected are appropriate. ED visits, hospital admissions and hospital days will be collected electronically from the Enterprise data warehouse (EDW) from Intermountain Healthcare (IH) and Lurie Children’s hospital (LCH), as well as Utah All Payer Claims Data (APCD) and Chicago CAPriCORN data. These data sets include strong data quality check and validation. Survey data that will also be collected from parents (or primary caregivers) via the REDCap, which also has data quality check. Surveys | **12** |
| Statistical methods | 27a | **Statistical methods used to compare groups for primary and secondary outcomes, including harms**  Analyses will be based on intention-to-treat by randomized assignment, irrespective of adherence. We will use descriptive statistics with means/standard deviations or medians/interquartile ranges for continuous variables, and frequencies/percentages for categorical variables to compare baseline characteristics between the study arms. We will compare the rate of ED/hospital admissions at 6 months post-enrollment between the two arms using Poisson or negative binomial regression with dispersion, possibly augmented to account for 0-inflation if the proportion or subjects with 0 ED events or hospitalizations exceeds that accounted for by the model. We will compare child QOL total score between the two arms at baseline, 3 and 6 months, using a linear mixed-effect model (LMM), with a random intercept to account for correlation in repeated measurements in the same patient. Adjustment for baseline QOL total score will account for any baseline imbalances in the outcome and regression to the mean. Also, secondary contrasts will estimate the treatment effect on the mean total score at each follow-up visit and compare treatment effect sizes between different follow-up visits to assess if the treatment effect attenuates or increases over time. We will compare other secondary outcomes (hospital days, parent stress, self-efficacy, and satisfaction) between the 2 arms using LMMs for numeric outcomes.  MyChildCMC use adherence will be determined as the number of logins with complete vital sign/ symptom recordings (out of all opportunities) over the first 6 months (months 1-6 or Period1) and second 6 months (months 7-12 or Period2) at individual parent level, and categorize parents into high (>50% logins) and low (≤50% logins) users (See Aim 2). For sustainability, we will compare parent level logins performance between Period1 and Period2, and categorize parents as sustainer (if MyChildCMC use adherence at 12 months (or end of Period2) remains at or is above level achieved at end of Period1), otherwise non-sustainer. We will assess sustainability of the intervention effect by comparing outcomes (ED/hospital admissions, total hospital days, QOL scores, parent stress scores, parent self-efficacy scores) between Periods1 and 2. | **4,5 16, 17** |
|  | 27b | **Definition of who will be included in each analysis (e.g., all randomized participants), and in which group.** Analyses will be based on intention-to-treat by randomized assignment irrespective of adherence. Therefore, analyses (Primary Objective) will include all randomized participants. | **4,5, 16, 17** |
|  | 27c | **How missing data will be handled in the analysis**  All analyses will utilize available data for all participants, irrespective of adherence to the intervention. For LMMs, participants who missed measures (primary and/or secondary outcomes) at certain but not all assessment time points will be included in the analyses, which will be performed using restricted maximum likelihood estimation and will therefore remain approximately unbiased in the presence of missing data as long as the pattern of missingness is random. If the proportion of missing data is >20%, we will use inverse probability of censoring weights or multiple imputation (with mixed-effect pattern-mixture models) to limit the effects of missing data. There may still be missingness in the outcome (ED/hospitalizations) due to limitations of data warehouses used, although it is likely a small percent based on preliminary data. We are combining site-specific with regional data (All Payer/Utah; CAPriCORN/Chicago), helping us to capture essentially all utilization data. | **19** |
|  | 27d | **Methods for any additional analyses (e.g., subgroup and sensitivity analyses)**  Covariates for the study are parent socio-demographics (age, race/ethnicity, marital status, education, household income) and social support score, child’s age, insurance (commercial/public), total number of caregivers, *MyChildCMC* use adherence, and child’s disability severity (FSS). Note: Some CMCs are cared for by multiple caregivers. Thus, we will do sub-analyses to assess if having multiple caregivers improves/worsen outcomes. Also, site differences of participants’ race/ethnicity may impact generalizability of our results. Thus, we will include site (SLC/Chicago) as random-effects in the comparative analyses to adjust for between-site differences. Further, since sites are referral centers for CMC from surrounding states, results can be generalized to CMC in multiple states. The possibility that the intervention effect differs based on the child’s and caregiver/parent’s **sex,** and child’s age groups (1-12 and 12-18) will be evaluated as a biological variable [Note: Child’s age and disability severity are part of covariates and used as potential effect moderators in Aims 1-3 as child and parent outcomes can be influenced by child disability severity and impairment duration (parents gaining experience over time)]. | **16, 17** |
| **Methods: Monitoring** | | |  |
| Data monitoring committee | 28a | **Composition of data monitoring committee (DMC); summary of its role and reporting structure; statement of whether it is independent from the sponsor and funder; conflicts of interest and reference to where further details about its charter can be found, if not in the protocol. Alternatively, an explanation of why a DMC is not needed**. We have an existing IRB approval (IRB_00088596) that supported the pilot trial for the current study. The application for the pilot trial was approved by the University of Utah IRB as a low-risk intervention. As in our pilot trial, we are not anticipating any adverse events that would harm study participants. The University of Utah single IRB classified the study as a minimal risk study. Therefore, we will use an Independent Safety Monitor (ISM) rather than a DMC or DSMB to monitor potential adverse events that may arise to participants during the study as DMC is not required. Dr. Justin Alvey is acting as the ISM for this project. Dr. Alvey, is a Professor of Pediatrics at the University of Utah. He is also the Vice Chair of the Pediatrics Clinical Enterprise Department, Chief, Division of General Pediatrics, and the Medical Director of the Primary Children's Pediatric Ambulatory Specialty Services. He has expertise in children with CMC care and research, and he is independent from the current study (not involved with the study). He will monitor, review and recommend appropriate action regarding adverse events and other safety issues. The ISM role is to oversee the safety of our intervention and assure the highest degree of study participant safety and data integrity during the trial. He will:   - Review the protocol as funded and make suggestions for any safety-related changes. - Establish guidelines for safety monitoring, including events that should be reported immediately. - Review study progress including recruitment, enrollment, retention, adherence (and protocol violations) and data quality. - Review proposed modifications to the study prior to their implementation and make recommendations. - Review and report SAEs. - Review AEs for safety and make recommendations as to whether the trial should continue as, be changed, suspended, or terminated based on the observed beneficial or adverse effects. - Submit written reports and suggestions to the PI and the NINR. - Meet with the study team to discuss and address safety concerns. | **19-22** |
|  | 28b | **Explanation of any interim analyses and stopping guidelines, including who will have access to these interim results and make the final decision to terminate the trial.** The recommended sample size is needed to achieve adequate power to detect the differences between the intervention and control groups. No interim analysis is needed prior to reaching the planned sample size. | **N/A** |
| Trial monitoring | 29 | Frequency and procedures for monitoring trial conduct. If there is no monitoring, give explanation | **N/A** |
| **Ethics** | | |  |
| Research ethics approval | 30 | **Plans for seeking research ethics committee/institutional review board approval.** The study was approved by the University of Utah IRB which is also acting as the Single IRB (sIRB) for both sites. Approval of all protocols, forms, informed consents, recruitment materials, assessment materials, and study modifications will be obtained from the sIRB before any enrollment. All subsequent amendments to the protocol, informed consent documents, and other materials will also be reviewed and approved by the SIRB prior to implementation. All SAEs, protocol violations, other reportable events and continuing reviews will also be submitted to the SIRB. | **20** |
| Protocol amendments | 31 | **Plans for communicating important protocol modifications to relevant parties**  No changes to the research protocol will be implemented without prior approval of the SIRB. We will communicate potential change to the protocol to the IRB during continuing review or amendment. And if approved, this information will be communicated to both sites and study staff, and then implemented only after approval. A Certificate of Action (approval letter) will be sent with each submission to each site, outlining the contents, dates of approval/expiration and instructions. | **20** |
| Consent or assent | 32a | **Who will obtain informed consent or assent from potential trial participants or authorized proxies, and how**. Clinical Research Coordinators | **2, 5, 6 ,14** |
|  | 32b | **Additional consent provisions for collection and use of participant data and biological specimens in ancillary studies, if applicable** | **N/A** |
| Confidentiality | 33 | **How personal information about potential and enrolled participants will be collected, shared, and maintained in order to protect confidentiality before, during, and after the trial.**  We will install rigorous security protections on data management, issuing regular reminders to project staff to change their passwords often, and adherence to HIPAA and IRB privacy and data security requirements will mitigate these risks. Approval of all protocols, forms, consents, and study modifications will be obtained from the single University of Utah’s IRB. Participants will be told in their consent process that they may choose not to answer any survey questions that upset them, and that they are free to withdraw from the study at any time. Confidentiality will be strictly maintained throughout the study. After consenting to participate, each patient will be assigned a unique study identification number. Research Coordinators (RCs) will maintain a cross-walk linking study IDs with patients' names. Patient names, phone numbers, emails and addresses will be required for recruitment and surveying. Data will be stored on a secured network directory at the University of Utah with authentication and authorization protections accessible only to the research team. Claims data from APCD will involve patient identifiers (including patient’s name, date of birth, address and gender) to link patients with claims data. Once patient encounters have been identified and the data retrieved, it will be de-identified for analysis, although a key will be maintained to ensure data integrity, which will be stored on a secured network directory at the University of Utah as stated above.  The principal risks to participants of this research are loss of privacy and confidentiality, including use of electronic databases for potential subject identification, and potential breach of confidentiality through disclosure of protected health information for parents responding to the ongoing assessment of crosscutting symptoms. Installing rigorous security protections on data management, issuing regular reminders to project staff to change their passwords often, and adherence to HIPAA and IRB privacy and data security requirements will mitigate these risks. Other risks may be overuse (increase ED/hospital admissions or healthcare encounters) driven by a significant change in vital sign/symptoms but with benign changes in actual child’s health status. *MyChidCMC* data is stored behind the University of Utah’s firewall within a HIPAA compliant database (HeidiSQL) located at the CHPC. Text/email alert messages are encrypted, and sent to the subjects free of protected health information, without patients’ names or any other identifiers, and the content will be very unlikely to pose a risk to confidentiality in the unlikely event that they were intercepted. Should new information become available during the study that may indicate that the risks of harm to participants have increased significantly, the investigators will inform participants, so they may reconsider their willingness to stay in the study. For the interviews, participants will receive initials or pseudonyms that will be used to link their responses to questions to protect their privacy. Data obtained from the surveys will be stored on a secured network directory with authentication and authorization protections accessible only to the research team. Reports generated by the *MyChildCMC* application including patient-specific clinical information will be accessible to parents through a HIPAA compliant and secured portal within the University of Utah’s firewall that includes authentication and authorization protections. We will use patient identifiers (e.g. name, date of birth, address and gender) to link patients with Intermountain and LCH electronic data to retrieve all ED/ hospital admissions to any affiliate Intermountain and LCH/CAPriCORN hospitals that occur during the study period. Once patient encounters have been identified, linked to the electronic data and ED/Hospital admission data as well as cost data retrieved, the combined data set will be de-identified for analysis with a key retained in a separate storage location. Data will be stored on a secured network directory at the University of Utah with authentication and authorization protections accessible only to the research team. All other research materials will be stored in a secure file cabinet in the PI’s secured office at PCH. The key to linking data back to PHI will be secured on the PI’s computer and will not be shared. All data analyses will be performed on data sets created without identifiers. Identifiers will be removed from study data files as early as possible in the data processing steps. All source/legacy data files will be stored behind the University of Utah firewall. All investigators and project staff will sign confidentiality pledges annually and receive annual IRB and HIPAA privacy and data security compliance training. | **20-24** |
| Ancillary and post-trial care | 34 | **Provisions, if any, for ancillary and post-trial care, and for compensation to those who suffer harm from trial participation** | **N/A** |

*We strongly recommend reading this checklist in conjunction with the SPIRIT 2025 Explanation and Elaboration and the SPIRIT 2025 Expanded Checklist for important clarifications on all the items. We also recommend reading relevant SPIRIT extensions. See [www.consort-spirit.org](http://www.consort-spirit.org)

Citation: Chan A-W, Boutron I, Hopewell S, Moher D, Schulz KF, et al. SPIRIT 2025 statement: updated guideline for protocols of randomised trials. BMJ 2025;389:e081477. <https://dx.doi.org/10.1136/bmj-2024-081477>

© 2025 Chan A-W et al. This is an Open Access article distributed under the terms of the Creative Commons Attribution License (<https://creativecommons.org/licenses/by/4.0/>), which permits unrestricted use, distribution, and reproduction in any medium, provided the original work is properly cited.
